# RB1CC1-enhanced autophagy facilitates PSCs activation and pancreatic fibrogenesis in chronic pancreatitis

**DOI:** 10.1038/s41419-018-0980-4

**Published:** 2018-09-20

**Authors:** Le Li, Gang Wang, Ji-Sheng Hu, Guang-Quan Zhang, Hong-Ze Chen, Yue Yuan, Yi-Long Li, Xin-Jian Lv, Feng-Yu Tian, Shang-Ha Pan, Xue-Wei Bai, Bei Sun

**Affiliations:** 1 Department of Pancreatic and Biliary Surgery, The First Affiliated Hospital of Harbin Medical University, Harbin, Heilongjiang China; 2 Department of Cardiology, The First Affiliated Hospital of Harbin Medical University, Harbin, Heilongjiang China; 3 Key Laboratory of Hepatosplenic Surgery, Ministry of Education, Department of General Surgery, The First Affiliated Hospital of Harbin Medical University, Harbin, Heilongjiang China

## Abstract

Chronic pancreatitis (CP) is described as a progressive fibro-inflammatory disorder of the exocrine disease, which eventually leads to damage of the gland. Excessive activation of pancreatic stellate cells (PSCs) is a critical participant in the initiation of CP. Autophagy is involved in multiple degeneration and inflammation in acute pancreatitis and CP. In our study, we report that retinoblastoma coiled coil protein 1 (RB1CC1) expression and the autophagic level are elevated in activated PSCs. RB1CC1 is positively correlated with pancreatic fibrogenesis in tissues and plasma of CP patients. Knockdown of RB1CC1 restrains alpha smooth muscle actin (α-SMA) and collagen expressions, and autophagy in activated PSCs in vitro. Furthermore, we show that RB1CC1 induces PSC activation via binding to ULK1 promoter and the direct interaction with ULK1 protein. These suppress ULK1 expression and its kinase activity. In mice, knockdown of RB1CC1 blocks autophagy and then inhibits the pancreatic duct ligation-induced pancreatic fibrosis. Consequently, our study highlights that RB1CC1-mediated autophagy is a key event for the activation of PSCs. Inhibition of RB1CC1 alleviates autophagy, which plays a critical role in anti-fibrotic activation in PSCs and CP progression. RB1CC1 could be a novel strategy for the treatment of pancreatic fibrosis.

## Introduction

Chronic pancreatitis (CP) has a prevalence of nearly 50/100,000 individuals^1,2^. It describes as wide spectrum of a fibro-inflammatory disease characterized by severe pain, development of fibrosis, and progression of endocrine and exocrine insufficiency. Prominent pathological features in CP involve acinar cell atrophy, chronic inflammation, block pancreatic ducts, and pancreatic stellate cell (PSC) activation^1–3^. Currently, no treatment has been shown to be effective in halting the fibrogenic process in CP.

PSCs are the main cell type in the stroma of CP and responsible for the regulation of the synthesis and degradation of extracellular matrix (ECM) proteins. In health, PSCs are located in close proximity to the basal aspect of pancreatic acinar cells and maintain the structure of normal pancreatic tissue. In response to pancreatic injury, quiescent PSCs are transformed into myofibroblast-like cells, which express α-smooth muscle actin (α-SMA). The α-SMA is a marker of activated PSCs and it could be able to promote PSC activation^4–6^. Activated PSCs proliferate, migrate, and produce ECM components, such as collagen, fibronectin matrix metalloproteinases (MMPs), as well as stimulate cytokines and chemokines production^4,5^. Activation of PSCs could occur by both autocrine and paracrine mechanisms, which hint that the effects of PSC activation, primarily inflammation, and resultant fibrosis could further progress, even after removing the primary source^6,7^. A large variety of growth factors (epidermal growth factor, platelet-derived growth factor, and vascular endothelial growth factor), cytokines (interleukin (IL)-1, IL-6, IL8, transforming growth factor-β (TGF-β), and tumor necrosis factor-α), and chemokines have been shown to activate PSCs in vitro^8,9^. Other known activators include alcohol, endotoxin, oxidant stress, hyperglycemia, pressure, protease, and factors pertinent to pancreatic injury^7–9^. The activation and functions of PSCs are transformed by the dynamic and coordinated intracellular biological processes. Therefore, elucidation of the molecular mechanisms which play a pivotal role in PSC activation may develop novel therapeutic approaches to minimize or reverse the pancreatic fibrosis.

Autophagy is a conserved cellular process that utilizes lysosomes-mediated degradation to turn over cellular proteins or organelles. It serves as an adaptive role to remove damaged organelles and cytoplasmic aggregates against diverse pancreatic disease, including acute pancreatitis, pancreatic cancer, and CP^10–12^. Autophagy may modulate PSC remodeling in the progression from a quiescent to an activated phenotype. Recent studies suggest that CP development appears to involve defects in autophagy and PSCs remain the autophagic competent of CP, which is required for initiation of fibrosis^13^. Xu et al.^14^ uncover a mechanism of TGF-β-induced PSC activation and ECM formation via inhibiting the PTEN/Akt/mTOR pathway. mTOR-induced autophagy is proved to be the core factors of reprogramming the hypersecretory phenotype in PSCs. In addition, Rottlerin is shown to alter autophagic and UPR signaling, cell fate, and fibrogenic and inflammatory responses in activated PSCs^15^. Moreover, ATG5-deficient mice have signs of endocrine insufficiency and exocrine pancreatic tissue destruction. ATG5 plays a critical role in CP through increasing the production of reactive oxygen species (ROS)^16^. However, the role of autophagy in CP is controversial. Accumulation of bile acid suppresses autophagy in pancreatic acinar cells signals via elevating the expression of nuclear farnesoid X receptor (FXR). FXR diminishes the expression of ATG7 and thereby triggeres inflammation and fibrosis in CP^17^. Besides, tocotrienol-rich fraction activates autophagic death in activated PSCs. The potential anti-fibrogenic value of tocotrienols may ameliorate the fibrogenesis by inducing autophagy and targeting the mitochondrial permeability transition pore^18^.

Retinoblastoma coiled coil protein 1 (RB1CC1) is also known as FIP200 (FAK family-interacting protein of 200 kDa) and is identified as a potential mammalian counterpart of autophagy protein Atg17. RB1CC1 is a component of the ULK1-ATG13-RB1CC1/RB1CC1-ATG101 complex and shown to be essential for autophagosome formation^19,20^. RB1CC1 could also regulate intracellular signaling pathways via interacting with and regulating different proteins, such as TSC1, p53, and PIASy^21–23^. All of them are involved in the functions of protein synthesis, cell proliferation, differentiation, migration, and cell cycle progression. Moreover, recent studies showed that several miRNAs, such as miR-20 and miR-224, regulate autophagy via targeting RB1CC1 in breast and cervical cancer cells^24,25^.

Given the proposed role of mRNAs in the regulation of activated PSCs and the relationship between PSC activation and pancreatic fibrosis, we conducted RNA sequencing to identify the agents that differentially modulate in PSC activation and their phenotypic expression. We also sought to characterize the mechanism behind how the dysregulation of RB1CC1 expression could affect the autophagic activity in PSCs and pancreatic fibrosis. Elevated RB1CC1 expression is the major contributor to PSC activation and increases pancreatic fibrosis progression through augmenting autophagy. Our findings open up new avenues for the use of RB1CC1 as a potential biomarker or molecular target in the development of diagnostic and therapeutic strategies for CP.

## Materials and methods

### Clinical samples and plasma

Tissues of 13 normal and 15 CP patients and plasma of 15 normal individuals and 15 CP patients were obtained from the First Affiliated Hospital of Harbin Medical University. The informed consent was obtained from patients and the study was approved by the review committee of the First Affiliated Hospital of Harbin Medical University.

### Cell culture

Mice PSCs in 2–3 passages were purchased from Cell Bank of Chinese Academy of Science. The PSCs kept the characteristics of primary cells. In our study, the passage times of PSCs were about 3–5 days used for each experiment and less than 15 passages of PSCs were used. Mice acinar cells (MPC-83) were purchased from Cell Bank of Chinese Academy of Medical Sciences and Peking Union Medical College. Mice islet β-cells (NIT-1) were purchased from American Type Culture Collection (CRL-2055). PSCs and NIT-1 were cultured in Dulbecco’s modified Eagle's medium (Gibco, Gaithersburg, USA) and MPC-83 was cultured in 1640 (Hyclone, Logan, USA), supplemented with 10% fetal bovine serum (Gibco), 1% penicillin and streptomycin at 37°C with 5% CO_2_.

### PSC isolation

Primary PSCs were isolated from murine pancreas by 0.03% collagenase P (Roche, Germany) digestion and Nycodenz density gradient centrifugation. Primary PSCs were maintained in Dulbecco’s modified Eagle's medium supplemented with 10% fetal bovine serum. The medium was changed and the contaminating cells were removed at 24 h after seeding. Primary PSCs were used before the first passage.

### cDNA library preparation and illumina sequencing for transcriptome analysis

Total RNA of PSCs and TGF-β-treated PSCs was extracted using Trizol reagent (Invitrogen, Carlsbad, USA) following the manufacturer’s protocol. RNA integrity was confirmed by the 2100 Bioanalyzer (Agilent Technologies). The samples for transcriptome analysis were prepared using Illumina’s kit following the manufacturer’s recommendations. The cDNA library was sequenced on the Illumina sequencing platform (HiSeqTM 2000). The raw reads from the images were generated. After removal of the low-quality reads, processed reads with an identity value of 95% and a coverage length of 100 bp were assembled using SOAP2 *de novo* software. The clean reads were assembled with the Trinity program, and the Trinities were clustered using TGICL tools into unigenes. The unigenes were used for BLAST search and annotation against an NCBI database using an *E*-value cut-off of 10^−5^. Functional annotation by gene ontology terms (GO, http://www.geneontology.org) was analyzed by Blast2Go software. The KEGG pathways annotation was performed using BLAST against Kyoto Encyclopedia of Genes and Genomes database (KEGG).

### Mice

Eight-week-old male C57BL/6 mice were purchased from the Experimental Animal Center of The Second Affiliated Hospital of Harbin Medical University (Harbin, China). Mice were randomly divided into five groups (negative control, CP, sh-RB1CC1, CP plus sh-negative control, and CP plus sh-RB1CC1). CP was induced by ligating the pancreatic duct at the junction of common duct and pancreatic duct. The bile duct and concomitant artery were spared. The mice received a single intraperitoneal injection of cerulein (50 mg/kg/body weight) 2 days after the pancreatic duct ligation^26^. In the sh-RB1CC1, CP plus sh-negative control and CP plus sh-RB1CC1 groups, the mice received a single intraperitoneal injection of lentiviral vector of negative control or sh-RB1CC1 at the second day after ligation. All of the mice were killed at 7, 14 or 21 days after ligation^27–29^. This study protocol was approved by the Institutional Review Board of The First Affiliated Hospital of Harbin Medical University.

### Hematoxylin and eosin staining, Masson staining, and immunohistochemistry

The hematoxylin and eosin (H&E) staining and immunohistochemical staining protocols were described previously^30,31^. Masson staining was performed following the manufacture’s protocol (Baso, Zhuhai, China). In the immunohistochemical staining assay, the paraffin-embedded tissue sections were immunostained with anti-RB1CC1, anti-Collagen I, anti-Collagen III, anti-α-SMA, anti-LC3B, anti-ULK1, anti-Beclin1, and anti-LAMP-2.

### Reagents and chemicals

TGF-β was purchased from Peperotech (Rocky Hill, USA). Chloroquine (CQ), Rapamycin (RAPA), and cerulein were ordered from Sigma (Sigma-Aldrich, Shanghai, China). pcDNA3.1-RB1CC1 was obtained from the Youbio (Changsha, China). si-negative control and si-RB1CC1 were obtained from Santa Cruz Technology (Dallas, USA). Lentiviruses of sh-negative control and sh-RB1CC1 were purchased from Genechem (Shanghai, China). The sequences of sh-RB1CC1 were listed as following:

sh-RB1CC1–1: CCAAGAAGCTCTGCTCTTT

sh-RB1CC1–2: GCTGTTGTTGAGGTTGTAA

sh-RB1CC1–3: GCAGAGGATCATGCTCCTA

### mRFP-GFP-LC3 assay

The mRFP-GFP-LC3 assay was performed as described previously^11,32^. PSCs were transfected with negative control and GFP-mRFP-LC3 lentiviral vector. The stably transfected cells were transfected with si-RB1CC1 and/or treated with TGF-β and were viewed under a fluorescence microscope. The number of GFP and mRFP dots was determined by manual counting of the fluorescent puncta in five high-power fields (original magnification, ×40, Olympus, Japan).

### Co-IP assay

The cells (1 × 10^7^) were lysed with 500 µl of lysis buffer (100 mM KCl, 5 mM MgCl_2_, 10 mM Hepes pH 7.0, 0.5% NP-40, 1 mM dithiothrectol, and Cocktail (Roche, Switzerland) for 30 min on the ice. The cell lysates were collected following centrifugation. The protein–protein immunocomplexes were formed by incubating 500 µl of cell lysates with 5 μg of anti-RB1CC1, anti-ULK1, and isotype control IgG (Sigma, St Louis, USA) at 4°C overnight and were brought down by 20 μl of protein A/G agarose beads (Millipore, Billerica, USA). After the beads were washed, the complexes were boiled 10 min in 100 ℃ and loaded on the gel.

### RNA isolation, reverse-transcription and quantitative real-time polymerase chain reaction (qRT-PCR)

RNA isolation and the PCR amplification conditions were followed as previously described^33^. qRT-PCR assay (SRBY Green) was performed on Applied Biosystem 7500. The relative expression levels of mRNAs were calculated and quantified using the 2^−^^ΔΔCT^ method. GAPDH served as the endogenous control. The primer sequences were designed by Primer 5.0 and are listed in Supplementary Materials.

### Transmission electron microscope

Transmission electron microscopy was performed as described previously^34^. Fresh tissues were fixed in 2.5% glutaraldehyde, and postfixed in 1% osmium tetroxide buffer. Tissues were embedded in spur resin and thin sections were cut. The sectioned grids were stained with a saturated solution of uranylacetate and lead citrate. Sections were examined at 80 kV using a JEOL 1200EX transmission electron microscope (Harbin Medical University, China).

### Immunofluorescence

Immunofluorescence was performed as described previously^35^. Primary PSCs transfected with si-RB1CC1 and the si-negative control were seeded on 24-well plates. The cells were fixed with 4% paraformaldehyde for 30 min and were permeabilized with 0.5% Triton X-100 for 20 min. After incubation for 2 h with anti-RB1CC1 (ProteinTech) and anti-α-SMA (Cell Signaling Technology), the cells were washed with PBS for three times. Then, the cells were incubated with secondary antibodies for 1 h (Beyotime, Nanjing, China), and 4′6-diamino-2-phenylindole (DAPI, Beyotime) was added to stain the cell nuclei. The cells were detected by a laser scanning confocal microscope (×40, Olympus, Japan).

### ULK1 promoter construction and Luciferase reporter assay

A 2000 bp DNA fragment with the predicted RB1CC1-binding site from the promotor of ULK1 (NC_000071.6) was cloned from mouse genomic DNA to the multiple cloning regions of the luciferase reporter pGL3-basic vector (Promega, Madison, USA). PSCs were co-transfected with the 0.3 μg of luciferase reporter vector plasmid, 0.1 μg pRL-CMV and 0.2 μg pcDNA3.1 encoding either RB1CC1 or si-RB1CC1. The Renilla luciferase containing pRL-CMV plasmid was used as a control for transfection efficiency. After 48 h, the cells were harvested and Firefly luciferase reporter assay and Renilla luciferase reporter assays were performed via the Dual-Glo luciferase assay system (Promega).

### Electrophoretic mobility shift assay

The putative RB1CC1-binding sites of ULK1 were analyzed by electrophoretic mobility shift assay (EMSA) according to the manufacturer’s instruction using the Chemiluminescent Nucleic Acid Detection Module Kit (Thermo Fisher Scientific, Hudson, USA). Oligonucleotides containing RB1CC1-binding site was synthesized with biotin at 5′ end. The RB1CC1-binding site for ULK1 was CTTTTTTTATAAAA CACAACA.

### Western blot analysis

Western blot analysis was performed as described previously^10,11^. Whole-cell lysates with approximately 40 μg of proteins were resolved on 10% and 12% SDS-PAGE and were subjected to western blot assay using the antibodies listed in Supplementary Materials. After appropriate secondary antibody incubation, the bands were visualized with the Molecular Imager System (BIO-RAD, Hercules, USA) using an enhanced chemiluminescence method (Thermo Fisher Scientific).

### Statistical analysis

Results are shown as the mean ± SD. Statistical analysis was performed with SPSS 19.0 software and analysis of variance (ANOVA) and a Student’s *t*-test were used to evaluate statistical significance. Differences are considered significant when *p< 0.05, **p < 0.01, ***p < 0.001 and ns *p* > 0.05.

## Results

### RB1CC1 expression is increased in activated PSCs

**Fig. 1:**
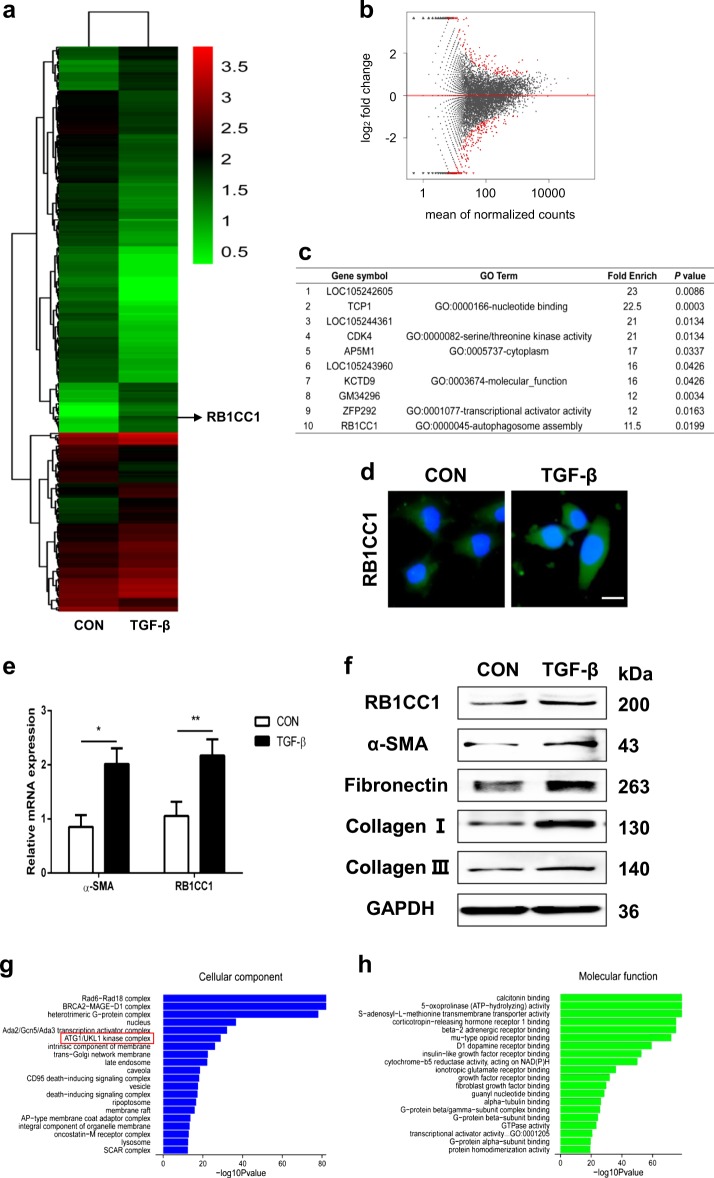
TGF-β induces RB1CC1 expression and accelerates PSC activation. **a**, **b** The heatmap and volcano plots showed that 433 genes were changed in activated PSCs compared with quiescent PSCs (fold change ≥2 or ≤0.5, *p* *≤* 0.05). **c** Top ten upregulated genes were listed and RB1CC1 was significantly elevated. **d**, **e** The expressions of RB1CC1 protein and mRNA were explored in quiescent and activated PSCs (Bars = 500μm). **f** Western blot analyses were detected to determine the expressions of RB1CC1, α-SMA, Fibronectin, Collagen I, and Collagen III in quiescent and TGF-β-treated PSCs. GAPDH served as the internal control. **g**, **h** Gene classification and gene function enrichment analysis of differentially expressed genes were performed (*p < 0.05 and **p < 0.01)

The characteristics of PSCs were detected before our experiment. The cell shape was monitored via a microscope and the expressions of RB1CC1, ULK1, p-ULK1, LC3, P62, and α-SMA were detected via western blot assays. We found that the PSCs kept the characteristics of primary cells and the expression of α-SMA was low. These indicated that the PSCs were non-activated or slightly activated (Supplementary Fig. 1). Previous studies have demonstrated that TGF-β was the main cause for PSC activation. Our data showed that the concentration of 10 ng/ml and incubation time of 24 h could reach the largest extent of PSC activation (Supplementary Fig. 2). The TGF-β concentration of 10 ng/ml and the time points of 24 h were used to incubate the PSCs in our study. To identify candidate mRNAs whose expressions are differentially altered in PSC activation and pancreatic fibrosis development, we initially examined the cDNA library of PSCs and TGF-β-treated PSCs on the Illumina sequencing platform. A total of 433 genes were changed in activated PSCs, including 168 upregulated genes and 265 downregulated genes (Fig. 1a, b and Supplementary Table 1). The top ten changed genes are listed in Fig. 1c. Our data revealed that RB1CC1 expression was significantly elevated in activated PSCs. RB1CC1 is a newly identified essential autophagy gene for maintaining protein homeostasis, attenuating autophagosome formation. Previous studies have showed that autophagy is required for activation of PSCs and over-activated autophagy exacerbates pancreatic fibrosis. We then performed the immunofluorescence and confirmed that expression of RB1CC1 was increased in activated PSCs (Fig. 1d). Furthermore, the qRT-PCR assay indicated that RB1CC1 and α-SMA mRNA expression were both elevated in TGF-β-treated PSCs (Fig. 1e). All of these revealed that the important biological function of RB1CC1 may participate in PSC activation. In addition, a series of indicators which could reflect pancreatic fibrosis, including Fibronectin, Collagen I, and Collagen III, were corelated with the upregulation of RB1CC1 in activated PSCs (Fig. 1f). Finally, the GO analysis revealed the top cellular component and molecular function for all the changed genes. The bioinformatic analyses demonstrated that the ATG1/ULK1 kinase complex is the main cellular component and most of them function as receptor or factor binding in activated PSCs (Fig. 1g, h and Supplementary Table 2).

### Autophagy accelerates PSC activation

**Fig. 2:**
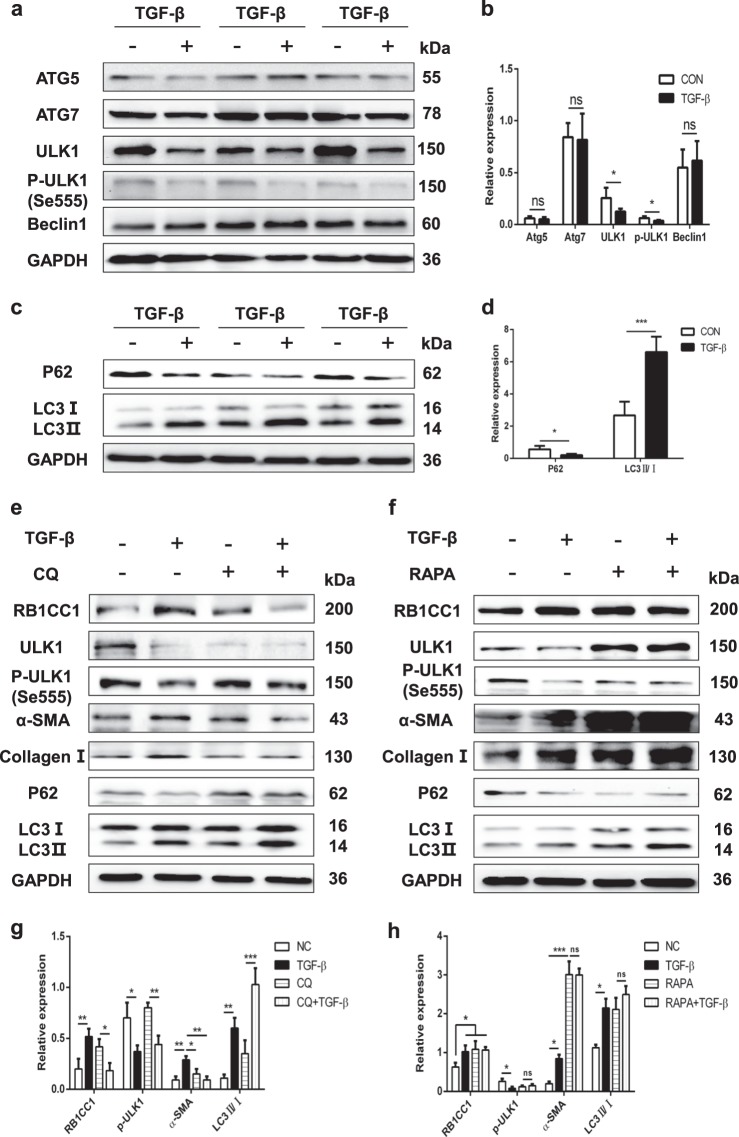
TGF-β inhibits the phosphorylation of ULK1 and promotes autophagy in activated PSCs. **a**, **b** The expressions of ATG5, ATG7, ULK1, p-ULK1 (Se555), and Beclin1 were tested in quiescent and activated PSCs. The relative expression represents the ratio of target to GAPDH. **c**, **d** The autophagic related indicators P62 and LC3 were examined via western blot assays. The relative expression represents the ratio of target to GAPDH. **e**, **g** The effects of autophagic inhibitor CQ (10μmol/L) was used to determine the RB1CC1 expression, autophagic levels (P62 and LC3), ULK1 expression and its kinase activity, and PSC activation (α-SMA and Collagen I) in quiescent, TGF-β-treated, CQ and TGF-β-treated plus CQ groups. **f**, **h** Role of autophagic activator RAPA (5μmol/L) was used to investigate the RB1CC1 expression, autophagic activation (P62 and LC3), ULK1 expression and its kinase activity, α-SMA and Collagen II in quiescent, activated, RAPA and activated plus RAPA groups. Data are expressed as mean ± SD. The results are representative of three independent experiments (*p < 0.05, **p < 0.01, ***p < 0.001)

As TGF-β treated PSCs activation play a critical role in CP, we examined the autophagic levels in control and TGF-β treated PSCs. The autophagic-related genes, including ATG5, Beclin1, ATG7, ULK1, and p-ULK1 were tested. Our data indicated that TGF-β strongly attenuated ULK1 expression and the kinase activity of ULK1 in PSC cell lines, but not the expressions of ATG5, ATG7, and Beclin1 (Fig. 2a, b). Next, we observed that TGF-β induced PSC autophagic activation via the inhibition of P62 and elevation of LC3II/I (Fig. 2c, d). As an autophagic inhibitor, chloroquine (CQ) restrains autophagy via blocking the fusion of autophagosome with lysosome. Our study found that CQ blocked PSC autophagy, but it could not suppress cells activation. Chloroquine had no impact on RB1CC1 expression and phosphorylation of ULK1 (Fig. 2e, g, Supplementary Fig. 3a). On the other hand, autophagic activator Rapamycin (RAPA) was found to enhance the RB1CC1 expression and PSC activation. It may increase autophagic activation via inhibiting the kinase activity of ULK1. Higher autophagic level consistently induces PSC activation (Fig. 2f, h, Supplementary Fig. 3b). Hence, we hypothesize that autophagy accelerates PSC activation in vitro and downregulation of the kinase activity of ULK1 stimulates autophagy, which is specially increasing in PSC activation.

### Knockdown of RB1CC1 attenuates autophagic flux and reduces PSC activation

**Fig. 3:**
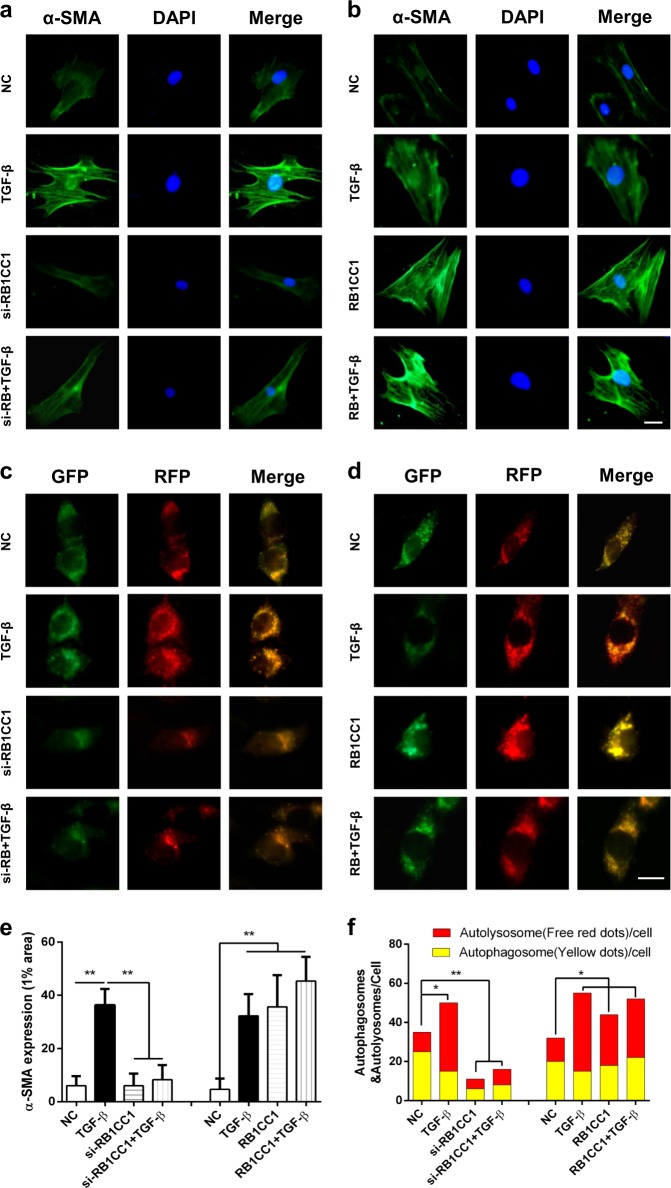
Knockdown of RB1CC1 inhibits autophagic flux and reduces PSC activation. **a**, **b** The α-SMA expression was explored in primary PSCs of quiescent, TGF-β-treated, RB1CC1-downregulated, si-RB1CC1 plus TGF-β, RB1CC1-upregulated, and pcDNA3.1-encoding RB1CC1 plus TGF-β groups via immunofluorescence staining (bars = 500μm). **c**, **d** The immunofluorescence assays were assessed in PSCs which were transfected with flag-tagged mRFP-GFP-LC3 lentiviral vector in eight different groups (bars = 500μm). **e** The α-SMA expressions in 1% area in different groups. **f** The numbers of RFP and mRFP dots were determined by fluorescent puncta in five high-power fields. Data are expressed as mean ± SD. The results are representative of three independent experiments (*p < 0.05 and **p < 0.01)

As primary PSCs may auto-activated in vitro culture and passages, we firstly explored the expression of RB1CC1 and autophagy machinery in the primary PSCs and PSCs. Our data indicated that the RB1CC1 expression and autophagic levels were nearly stable in less than 15 passages of PSCs compared with primary PSCs (Supplementary Fig. 1b). Furthermore, we screened knockdown of RB1CC1 on α-SMA expression to investigate its role in PSC activation. Our data indicated that sliencing of RB1CC1 downregulated the α-SMA expression and it also abolished the TGF-β-induced α-SMA accumulation (Fig. 3a, b, e, Supplementary Fig. 4). In addition, downregulation of RB1CC1 promoted cell proliferation, stabled cell vitality, and suppressed apoptosis in unactivated and activated PSCs (Supplementary Figs. 5 and 6). Restraint of RB1CC1 suppressed and even reversed migration, MMP-2 and MMP-9 production in non-treated and TGF-β-treated PSCs (Supplementary Figs. 7 and 8). It could abrogate the effects of TGF-β on PSC proliferation, apoptosis, migration, and ECM. As autophagic flux is a dynamic process, we then investigated the effect of RB1CC1 on the autophagic flux via mRFP-GFP-LC3 probe. Our data revealed that TGF-β caused increased yellow and red dots in merged images, indicating activation of both autophagosome formation and lysosome degradation in activated PSCs, whereas downregulation of RB1CC1 pretreatment resulted in accumulation of yellow dots, which implied impaired auto-lysosomal degradation (Fig. 3c, d, f). All of these suggest that RB1CC1 is an essential regulator of autophagy and it serves as an initiator and facilitator in PSC activation.

### RB1CC1 induces PSC activation via binding to ULK1 promoter and the direct interaction with ULK1

**Fig. 4:**
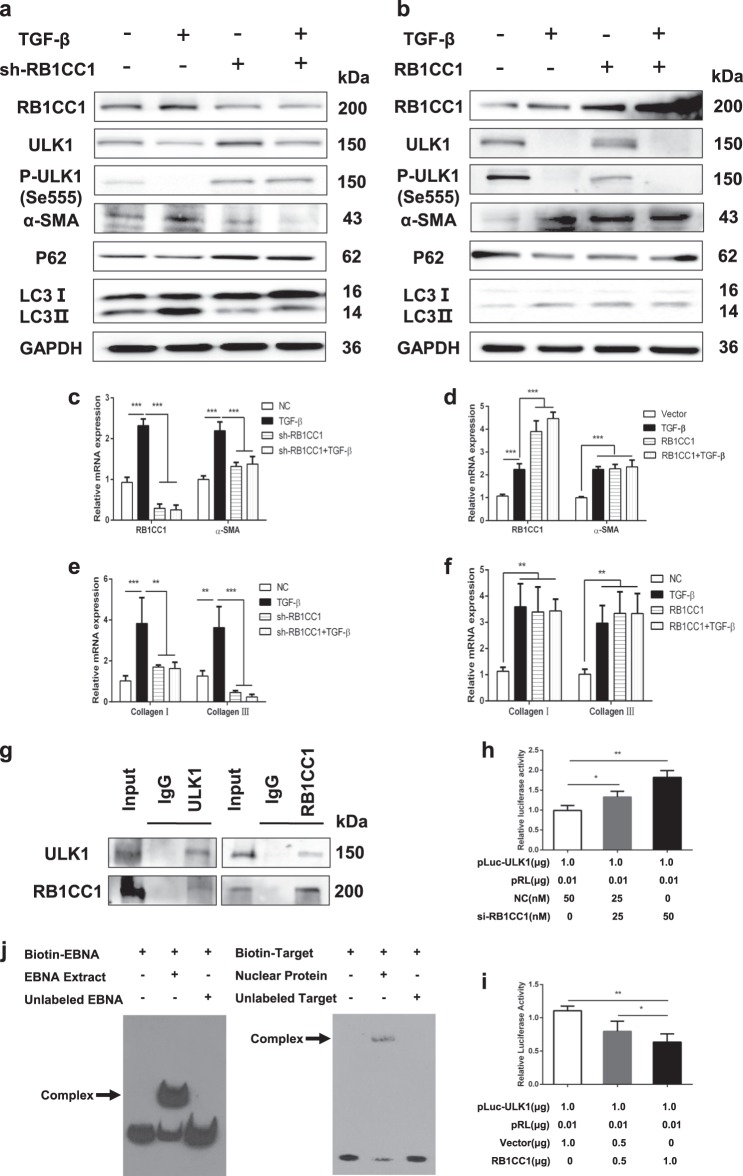
RB1CC1 induces PSC activation via binding to ULK1 promoter and the direct interaction with ULK1 protein. **a** Western blot analyses were assessed to reveal the expressions of RB1CC1, ULK1, p-ULK1, α-SMA, P62, and LC3II/I in quiescent, activated, sh-RB1CC1 and sh-RB1CC1 plus TGF-β groups. GAPDH served as the internal control. **b** The expressions of RB1CC1, ULK1, p-ULK1, α-SMA, P62 and LC3II/I were measured by Western blot assays in quiescent, TGF-β, pcDNA3.1 encoding RB1CC1 and pcDNA3.1 encoding RB1CC1 plus TGF-β groups. **c**, **d** The mRNA levels of RB1CC1 and α-SMA were tested by qRT-TCR assays in different groups. **e**, **f** The relative expressions of Collagen I and Collagen III were determined via qRT-TCR assays. **g** The Co-IP analyses were performed and the relative enrichments of RB1CC1 or ULK1 were determined by western blot assays in Input, IgG, and ULK1 or RB1CC1 groups. **h**, **i** Luciferase assays indicated that RB1CC1 could bind with ULK1 promoter and regulated its transcription. **j** Electrophoretic mobility shift assay (EMSA) was carried out using biotinylated DNA oligos containing the RB1CC1-binding site (CTTTTTTTATAAAACACAACA) in the ULK1 promotor. Data are expressed as mean ± SD. The results are representative of three independent experiments (*p <0.05, **p <0.01 and ***p <0.001)

RB1CC1 forms a complex with ATG1 and ATG13 and is essential for the autophagy regulation. ULK1-interacting proteins isolated RB1CC1 as an ULK1-binding partner, which is important for the autophagosomes formation.^36^ Our data showed that sliencing of RB1CC1 inhibited ULK1 expression and its kinase activity, and these blocked the autophagic activation and α-SMA expression. Downregulation of RB1CC1 abolished the increase of α-SMA, Collagen I and Collagen III expressions in TGF-β-treated PSCs (Fig. 4a, c, e, Supplementary Fig. 9a). Furthermore, overexpression of RB1CC1 adversely induced the formation of autophagy and PSC activation. However, we found lower expression of ULK1 and its kinase activity following the upregulation of RB1CC1 (Fig. 4b, d, f, Supplementary Fig. 9b). Upregulation of RB1CC1 enhanced α-SMA, Collagen I, and Collagen III expressions on the TGF-β-induced PSC activation. These results suggest that the decrease in ULK1 expression and phosphorylation of ULK1 due to the elevation of RB1CC1 contributes to the increase in α-SMA expression. ULK1 functions as an essential mediator for PSC activation. To explore the main mechanisms of RB1CC1 inhibiting ULK1 expression and its kinase activity, we searched the predicted functional partner and explored the protein–protein between RB1CC1 and ULK1 in PSCs. Our results confirmed the direct interaction between RB1CC1 and ULK1 protein via the co-immunoprecipitation assay (Fig. 4g). To further investigate the regulatory mechanism underlying the correlation between RB1CC1 and ULK1, the 2000 bp pGL3-ULK1 promotor was cloned and co-transfected into PSCs with pcDNA3.1 encoding RB1CC1 and si-RB1CC1. The luciferase reporter assay indicated that RB1CC1 may activate PSCs via binding with ULK1 promotor (Fig. 4h, i). Subsequently, three online software programs (Gene2promotor Tool, PROMO (http://alggen.lsi.upc.es/cgi-bin/promo_v3/promo/promoinit.cgi?dirDB=TF_8.3) and EpiTect ChIP qPCR Primers (http://www.sabiosciences.com/chipqpcrsearch.php?app=TFBS) were used to search the possible transcription factor-binding sites in ULK1 promotor. We predicted three putative RB1CC1-binding sites within 2000 bp upstream of the transcriptional start of ULK1 (Supplementary Fig. 10). The EMSA results indicated that RB1CC1 may directly bind with the Site 2 (TATAAAA) to suppress ULK1 transcription (Fig. 4j).

### Reducing RB1CC1 expression alleviates mice pancreatic fibrosis

**Fig. 5:**
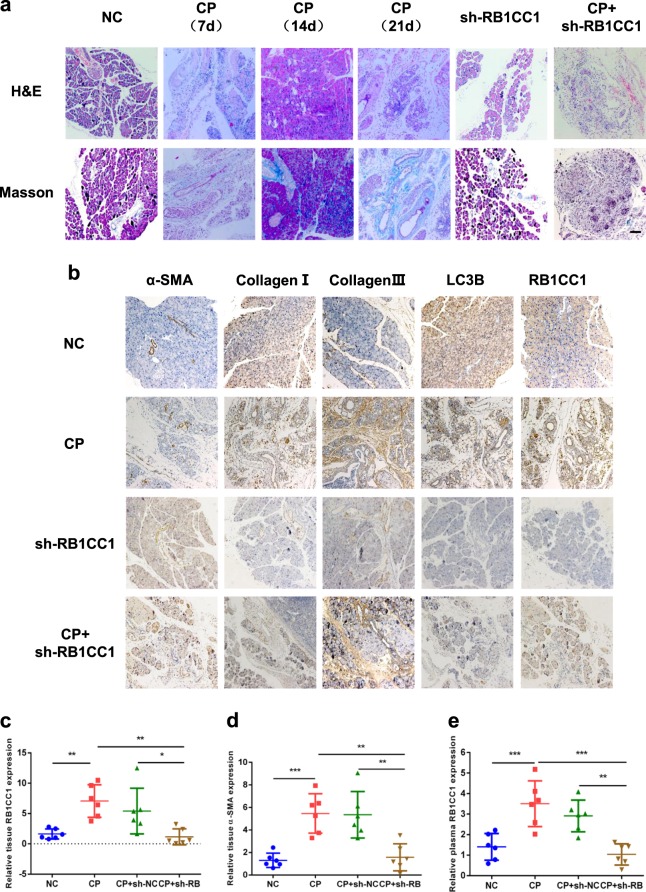
RB1CC1 promotes pancreatic fibrosis in CP mice. **a** The H&E and Masson assays were conducted to indicate CP and pancreatic fibrosis in mice models of sham, CP (7 d, 14 d, and 21 d), sh-RB1CC1, and CP plus sh-RB1CC1 groups (*n* = 6) (bars = 50μm). **b** The expressions of α-SMA, Collagen I, Collagen III, LC3B, and RB1CC1 were analyzed in four different groups (*n* = 6) (bars = 50μm). **c**, **d** The qRT-PCR assays were performed to determine the expressions of RB1CC1 and α-SMA in mice pancreas in negative control, CP (21 d), CP plus sh-NC injection, and CP plus sh-RB1CC1 injection groups (*n* = 6). **e** The plasma RB1CC1 levels were compared in four different groups (*n* = 6). Data are expressed as mean ± SD. The results are representative of three independent experiments (*p < 0.05, **p < 0.01, and ***p  < 0.001)

We generated the sh-RB1CC1 lentivirus to knockdown RB1CC1 expression in mice PSCs. Our aim was to confirm the role of RB1CC1 in PSC activation and to identify the possible treatment for pancreatic fibrosis. We found that knockdown of RB1CC1 prevented pancreatic fibrosis and delayed the improvement of CP (Fig. 5a). Sliencing RB1CC1 reduced the levels of α-SMA, Collagen I, Collagen III, LC3B, Beclin1, and LAMP-2 due to the inhibition of autophagic formation, while the expression of ULK1 was elevated (Fig. 5b). Besides, our data revealed that the level of RB1CC1 in plasma of CP mice was increased and intraperitoneal injection of sh-RB1CC1 lentivirus reduced RB1CC1 expression in plasma (Fig. 5c, d). To investigate the effects of RB1CC1 on other cells derived from pancreas, the western blot assays were performed in acinar cell line (MPC-83) and islet β-cell line (NIT-1). Our data showed that downregulation of RB1CC1 inhibited RB1CC1 and LC3II/I expressions, while the levels of P62, ULK1, and p-ULK1 were ascended. Additionally, RB1CC1 may promote the secretion of TGF-β in acinar cells (Supplementary Fig. 11). Taken together, the increase of RB1CC1 in PSCs is critical for developing pancreatic fibrosis and could function as a therapeutic target in the treatment of CP. Circulating RB1CC1 in plasma may be an effective indicator for monitoring CP progression (Fig. 5e).

### RB1CC1 expression correlates with pancreatic fibrosis in CP patients

**Fig. 6:**
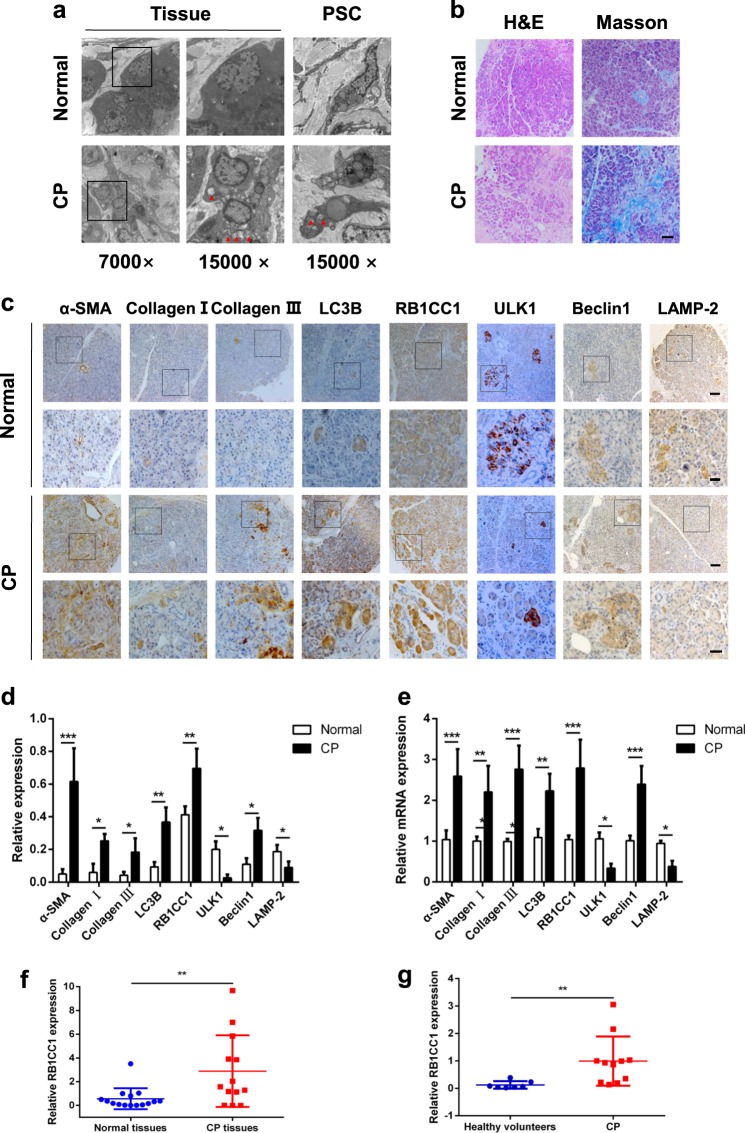
RB1CC1 expression is correlated with the severity of pancreatic fibrosis in CP patients. **a** The TEM assays revealed the autophagic levels in human normal tissues (*n* = 4) and CP tissues (*n* = 4), also in human quiescent and activated PSCs (*n* = 4). **b** The H&E and Masson assays were assessed to show the formation of fibrosis and collagen in normal tissues (*n* = 4) and CP tissues (*n* = 4) (bars = 50μm). **c**, **d** The expressions of α-SMA, Collagen I, Collagen III, LC3B, RB1CC1, ULK1, Beclin1, and LAMP-2 were analyzed via immunohistochemistry in normal tissues (*n* = 4) and CP tissues (*n* = 4) (bars = 50 μm, original magnification, ×10; bars = 20 μm, original magnification, ×40). The relative expressions of each indicator were analyzed in five high-power fields. **e** The expressions of α-SMA, Collagen I, Collagen III, LC3B, RB1CC1, ULK1, Beclin1, and LAMP-2 were determined via qRT-PCR assays. The relative expression represents the ratio of target to GAPDH. **f** The relative mRNA level of RB1CC1 was increased in CP tissues (*n* = 13) compared with normal tissues (*n* = 15). **g** The RB1CC1 mRNA was significantly elevated in plasma of CP patients (*n* = 11) than that in health individuals (*n* = 7). Data are expressed as mean ± SD. The results are representative of three independent experiments (*p < 0.05, **p < 0.01, and ***p < 0.001)

We examined autophagic levels in CP tissues. The results showed that the formation of autophagosome in clinical samples was increased as well as in PSCs (Fig. 6a). More fibrosis was observed, which indicated an abundant production of collagen in CP tissues (Fig. 6b). Immunohistochemistry revealed high levels of RB1CC1 in CP tissues and α-SMA, Collagen I, and Collagen III were also elevated compared with normal tissues. In addition, the expressions of LC3B and Beclin1 were higher than that in normal tissues, while the ULK1 and LAMP-2 level was lower in CP tissues. These indicated that increasing autophagic level is correlated with pancreatic fibrosis (Fig. 6c, d). The qRT-PCR results showed that the levels of α-SMA, Collagen I, Collagen III, LC3B, and Beclin1 mRNA were elevated while the level of ULK1 and LAMP-2 was declined (Fig. 6e). Furthermore, we found that RB1CC1 mRNA increased significantly in pancreatic fibrosis tissues and CP patients also have a higher RB1CC1 level in plasma (Fig. 6f, g). These suggest that RB1CC1-induced autophagy is considered as one possible mechanism for the exacerbation of pancreatic fibrosis. RB1CC1 may serve as an effective biomarker for pancreatic fibrosis.

## Discussion

Autophagy is a complex process that could be regulated by multiple signaling pathways and induced by many factors, including growth factors, amino acids, changement of glucose levels, and DNA damage^37–39^. It is also linked to the pathophysiological changes of pancreatic diseases, such as the damage of pancreatic acinar cell in acute pancreatitis, pancreatic fibrosis in chronic inflammation, and the complex cellular dysregulation in pancreatic cancer^40–42^. Although autophagic activation is critical to PSC activation and pancreatic fibrosis, the mechanism needs to be further explored. The widespread involvement of these highlights its potential as a target for the development of molecularly therapeutics in disease management. Taking advantage of RNA sequencing technology, a myriad of genes were found to be dysregulated in activated PSCs, which correlated with certain clinical characteristics, suggesting the novel roles in chronic inflammation progression. Our studies identified an essential autophagy-related gene RB1CC1 that plays an important role in PSC activation and pancreatic fibrosis.

RB1CC1 was identified as a component of ULK1/2 and Atg13 complex and showed to be essential for autophagy induction. Currently, there are much less studies to address RB1CC1 in chronic inflammation and fibrosis. Ma et al.^43^ found that RB1CC1 deficiency leads to progressive liver injury, fibrosis, and inflammation in nonalcoholic fatty liver disease. It was also found that RB1CC1 enhances proteasomal degradation of several negative regulators in the TGF-β signaling pathway via acting as a cofactor for the E3 ubiquitin ligase RNF111^44^. However, the function of RB1CC1 in PSC activation is not clear. Our study revealed that RB1CC1 was increased in activated PSCs and CP tissues. Upregulated RB1CC1 exacerbated pancreatic fibrosis through promoting the autophagic activation.

Our findings corroborate the hypothesis of the role of RB1CC1 in PSC activation based on the transcriptome sequencing. We found that RB1CC1 accelerates α-SMA and Collagen expression in activated PSCs. These indicated that the accumulation of RB1CC1 in PSCs may switch on or combust the pro-fibrotic signaling pathways. Previous studies have showed that autophagy induces PSCs remodeling in the progression from a quiescent to an activated phenotype^45^. We explored that the increment of α-SMA expression in activated PSCs is linked to the RB1CC1-medicated autophagy. Our data revealed that blockade of RB1CC1 could decrease the level of autophagy in vitro and in vivo. In addition, the expression of ULK1 and the phosphorylation of ULK1 was downregulated following the knockdown of RB1CC1. These results indicate that RB1CC1 accelerate autophagy in the activation of PSCs, which is relying on the restraint of ULK1 expression and its kinase activity.

ULK1 is named as autophagy-related gene 1 (ATG1) in yeast. It integrates signals from upstream sensors, such as mTOR and AMPK, and transduces them to the downstream autophagy pathway^45^. An increasing number of progresses have been made in understanding the mechanisms by which ULK1 is regulated through protein–protein interactions, translational and post-translational modifications. In our study, ULK1 and its kinase activity are significantly downregulated following the downregulation of RB1CC1. Several mechanisms have been explored between RB1CC1 and ULK1 in regulating autophagy. Hara et al.^46^ demonstrated that RB1CC1 is required for ULK1 puncta formation and is important for the stability and proper phosphorylation of ULK1. Hosokawa et al.^47^ showed that Atg13 directly interacts with ULK1 and mediates the interaction between ULK1 and RB1CC1. The ULK–Atg13–RB1CC1 complexes are direct targets of mTOR and important regulators of autophagy in response to mTOR signaling. Sullivan et al.^48^ corroborated that C9orf72/SMCR8/WDR41 associates with the RB1CC1–ULK1 complex, which is essential for autophagy initiation. In our present study, we firstly reported that RB1CC1 carries out its functions by binding to the promoter of ULK1 and inhibiting its transcriptional activity. We predicted three binding sites of RB1CC1 on the promoter region of ULK1 and the EMSA indicated that RB1CC1 may bind with the Site 2 to inhibit ULK1 transcription. It is particularly notorious that ULK1 kinase activity is subject to substantial post-translation modifications. ULK1 is hyperphosphorylated in nutrient and energy rich conditions and undergoes dephosphorylation upon starvation. The phosphorylation of ULK1 at different sites may have variety of functions in autophagy. Although more than 30 phosphorylation sites have been identified, the bulk of the responsible kinases and the effects of these phosphorylation events remain to be explored^49^. These imply that phosphorylation is a significant way of ULK1 regulation. Ser555, Ser637, and Ser757 were widely reported in the potential phosphorylation sites^50,51^. Our study found that RB1CC1 weakens the phosphorylation of ULK1 (Ser555) through the direct RB1CC1–ULK1 protein interaction. The dephosphorylation of ULK1 facilitates autophagy and then induces PSC activation. Besides, RB1CC1 may have other functions beyond stabilizing ULK1 and enhancing its kinase activity, such as acting as scaffolds for recruitment of other proteins and influencing the localization of the ULK1 complex. Currently, dephosphorylation of ULK1 upon autophagic activation requires more than blocking protein kinases activity such as mTOR, yet the protein phosphatase involved in weakening the kinase activity of ULK1 is unknown, let alone whether such a phosphatase is regulated in coordinated with other complex components. These findings highlight that dephosphorylation of ULK1 induced autophagy is a critical molecular mechanism for RB1CC1-induced PSC activation.

**Fig. 7:**
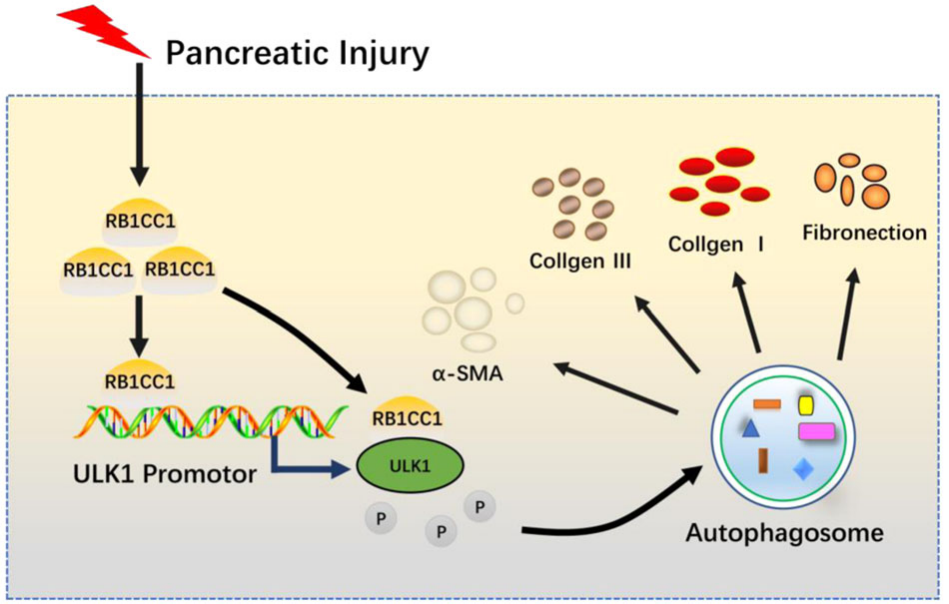
Schematic presentation of the mechanism underlying RB1CC1-facilitated PSC activation and pancreatic fibrosis. RB1CC1 bound to ULK1 promotor and represses its expression and kinase activity, which consequently induce autophagy and promote PSC activation and pancreatic fibrosis

PSC activation is a core process in pancreatic fibrogenesis. However, the target of suppressing PSC activation remains to be an unresolved issue. Our data suggest that RB1CC1 functions as a potential therapy to overcome pancreatic fibrosis in CP patients and mice models. Its expression is correlated with autophagic activation and pancreatic fibrosis in CP tissues. The effect of sh-RB1CC1 lentivirus indicated a significant amelioration in CP mice, which includes the improvement of multiple fibrosis-related markers, such as α-SMA, Collagen I and Collagen III, and autophagic-related gene, such as LC3. Moreover, CP induced higher RB1CC1 levels in the plasma of CP patients and mice and suppression of RB1CC1 reduced the circulating RB1CC1 expression in CP mice, which suggests that RB1CC1 could be used as a specific biomarker for monitoring pancreatic fibrosis. PSCs are present in islets and are involved in islet fibrogenesis in patients and animal models of type 2 diabetes (T2DM), which leads to progressive islet β-cell loss and dysfunction. Autophagy has been proposed to promote survival under conditions of β-cells stress that can lead to cell death, including nutrient depletion, oxidative stress, endoplasmic reticulum (ER) stress, mitochondrial damage, and hypoxia. Li et al.^52^ demonstrated that β-cells induce MMP-2 activity and reduce the production of ECM from PSCs. Hence, we speculate that RB1CC1-induced autophagy secretes more cytokines in β-cells which may promote the production of ECM and TGF-β in PSCs. Previous studies have demonstrated that lower autophagic level in acinar cells may accelerate CP progression via exacerbation of endoplasmic reticulum stress, accumulation of dysfunctional mitochondria, oxidative stress, activation of AMPK, and a marked decrease in protein synthetic capacity. In our study, we found that RB1CC1-induced autophagy in acinar cells may enhance TGF-β secretion which may accelerate PSC activation. As the PSCs have emerged as the major effector cells in CP, we believe that RB1CC1-induced TGF-β secretion in acinar cells functions as one of the key roles in pancreatic fibrosis. Together with these findings, RB1CC1-induced autophagy plays an important role in PSC activation and inhibition of RB1CC1 could be a prospective strategy to overcome pancreatic fibrosis (Fig. 7).

There is a potential limitation of this study. The PSCs used in this study was purchased from Cell Bank of Chinese Academy of Science. Although the cells kept some of the characteristics of primary cells, the fact that most of the experiments were not performed using primary PSCs. In summary, our study corroborated that RB1CC1 plays a key role in the dysregulation of PSC activation. We demonstrated that the ablation of RB1CC1 attenuates PSC activation through negatively affecting ULK1 expression and its phosphorylation. The RB1CC1–ULK1 complex as an essential component that connects RB1CC1 and autophagy in the activation or homeostasis of PSCs. RB1CC1 could be a critical target for the treatment of pancreatic fibrosis. These results provide novel insight into the regulation of PSC activation and pancreatic fibrosis by autophagy.

## Electronic supplementary material

Revised Sup material

Revised Supplementary Figure

Supplementary Table 1–433 differentially expressed genes

Supplementary Table 2-KEGG analysis

Supplementary Table 3

Supplementary Table 4
