# Supplementary material for: RB1CC1-enhanced autophagy facilitates PSCs activation and pancreatic fibrogenesis in chronic pancreatitis
Source: Cell Death Dis. 2018 Sep 20;9(10):952. doi: 10.1038/s41419-018-0980-4 (PMC6147947; doi:10.1038/s41419-018-0980-4)
Supplement: Supplementary file 1 — Revised Sup material [file 41419_2018_980_MOESM1_ESM.pdf]

RB1CC1-enhanced autophagy facilitates PSCs activation and pancreatic fibrogenesis in chronic pancreatitis

Supplementary Materials

1.Cell viability assay

Cell viability assay was performed by the Cell Counting Kit-8 (CCK-8) Kit (Dojindo, Tokyo, Japan) following the instructions described previously[1]. PSCs were plated in 96-well plates at a density of 3×10^3 cells per well. After treatment, CCK-8 solution (10μL) was added to each well and the plates were incubated at 37°C for 90 minutes. The absorbance of the cell suspension was measured at a wavelength of 450nm. Medium containing 10% CCK-8 solution served as a control.

2.EdU Retention Assay

EdU Retention Assay was performed as described previously[2]. PSCs were stain with EdU using the Cell-Light EdU DNA Cell Proliferation Kit (RIBOBio, Guangzhou, China). The cells were treated with 10μmol/L EdU for 2h at 37°C and fixed with 4% paraformaldehyde for 30min. Then, the cells were treated with 0.5% Triton X-100 for 20min. Next, the cells were exposed to 100μl 1×Apollo reaction cocktail for 30min and were stained with Hoechst 33342 for 30min. The PSCs were visualized with a fluorescent microscope (10×, Olympus, Japan).

3. Migration assay

Migration assay was performed as described previously[2]. PSCs were plated in 24-well BioCoat Matrigel Invasion Chambers (Corning, NY, USA). The cells on the top

surface of the filter were carefully removed with a cotton swab after 24 hours. The cell numbers on each membrane were counted in five high power fields using a microscope (20×, Olympus, Japan).

4. The primer sequences for qRT-PCR assays

RB1CC1 (Mice)

Upper Primer: 5' TGATTTGTCCTCCAGTGCTGA 3'

Lower Primer: 5' AGGTAAGTGCAGTCCACCAA 3'

α-SMA (Mice)

Upper Primer: 5' CATCCTGGCTTCGCTGTCTAC 3'

Lower Primer: 5' AAGGAACTGGAGGCGCTGAT 3'

Collagen Ⅰ (Mice)

Upper Primer: 5' GCTCCTCTTAGGGGCCACT 3'

Lower Primer: 5' ATTGGGGACCCTTAGGCCAT 3'

Collagen Ⅲ (Mice)

Upper Primer: 5' CTGTAACATGGAAACTGGGGAAA 3'

Lower Primer: 5' CCATAGCTGAACTGAAAACCACC 3'

GAPDH (Mice)

Upper Primer: 5' CTGCTCCTCCCTGTTCCAGAG 3'

Lower Primer: 5' CCCAATACGGCCAAATCCG 3'

RB1CC1 (Human)

Upper Primer: 5' CCTAGACGAACGCCATGACA 3'

Lower Primer: 5' AGCTGGTTTGAGATCCAGGG 3'

α-SMA (Human)

Upper Primer: 5' CTAGCACCCAGCACCATGAA 3'

Lower Primer: 5' TTGTACAAGAAAGTTGGTTAGAAGC 3'

ULK1 (Human)

Upper Primer: 5' GGCAAGTTCGAGTTCTCCCG 3'

Lower Primer: 5' CGACCTCCAAATCGTGCTTCT 3'

Collagen Ⅰ (Human)

Upper Primer: 5' GAGGGCCAAGACGAAGACATC 3'

Lower Primer: 5' CAGATCACGTCATCGCACAAC 3'

Collagen Ⅲ (Human)

Upper Primer: 5' GGAGCTGGCTACTTCTCGC 3'

Lower Primer: 5' GGGAACATCCTCCTTCAACAG 3'

LC3B (Human)

Upper Primer: 5' CTTACAGCTCAATGCTAATCAGG 3'

Lower Primer: 5' ACACTGACAATTTCATCCCGAAC 3'

Beclin1 (Human)

Upper Primer: 5' CCATGCAGGTGAGCTTCGT 3'

Lower Primer: 5' GAATCTGCGAGAGACACCATC 3'

LAMP-2 (Human)

Upper Primer: 5' GAAAATGCCACTTGCCTTTATGC 3'

Lower Primer: 5' AGGAAAAGCCAGGTCCGAAC 3'

GAPDH (Human)

Upper Primer: 5' CCTCTGACTTCAACAGCGACCAC 3'

Lower Primer: 5' TGGTCCAGGGGTCTTACTCC 3'

5.Antibodies for Western blot assays

**Antibody**	**Company**	**Dilution**
α-SMA	ProteinTech (14395-1-AP)	1:2000
Collagen Ⅰ	ProteinTech (14695-1-AP)	1:1000
Collagen Ⅲ	ProteinTech (22734-1-AP)	1:1000
Fibronectin	ProteinTech (15613-1-AP)	1:1000
BAX	ProteinTech (50599-2-IG)	1:2000
MMP-2	ProteinTech (10373-2-AP)	1:1000
MMP-9	ProteinTech (10375-2-AP)	1:1000
ATG5	Abcam (ab108327)	1:1000
ATG7	Abcam (ab133528)	1:1000
LC3	Cell Signaling Technologies (3868)	1:2000
P62	Cell Signaling Technologies (5114)	1:2000
Beclin1	Cell Signaling Technologies (3495)	1:2000
ULK1	Cell Signaling Technologies (8054)	1:1000
p-ULK1	Cell Signaling Technologies (5869)	1:1000
BCL-2	Santa Cruz Biotechnology (sc-7382)	1:1000
TGF-β	Invitrogen (MA5-16949)	1:2000
GAPDH	Kangcheng (KC-5G5)	1:4000
β-actin	Santa Cruz Biotechnology (sc-58673)	1:3000

Secondary antibodies	Santa Cruz Biotechnology (sc-2004, sc-2005)	1:10000

6.Antibodies for immunohistochemistry assays

**Antibody**	**Company**	**Dilution**
RB1CC1	ProteinTech (17250-1-AP)	1:200
ULK1	ProteinTech (20986-1-AP)	1:200
Collagen Ⅰ	ProteinTech (14695-1-AP)	1:200
Collagen Ⅲ	ProteinTech (22734-1-AP)	1:200
LC3B	Cell Signaling Technologies (3868)	1:200
α-SMA	Cell Signaling Technologies (19245)	1:200
Beclin1	Cell Signaling Technologies (3495)	1:200
LAMP-2	Invitrogen (PA1-655)	1:200

Reference

1. Wang Y, Zhou Y, Jia G, Han B, Liu J, Teng Y, et al. Shikonin suppresses tumor growth and synergizes with gemcitabine in a pancreatic cancer xenograft model: involvement of NF-κB signaling pathway. Biochem Pharmacol. 2014; **88**:322-333.
2. Li L, Chen H, Gao Y, Wang YW, Zhang GQ, Pan SH, et al. Long noncoding RNA MALAT1 promotes aggressive pancreatic cancer proliferation and metastasis via the stimulation of autophagy. Mol Cancer Ther. 2016; **15**:2232-2243.

Supplementary Figure Legend

Supplementary Fig. 1 The effect of PSCs auto-activation on RB1CC1 expression and autophagy machinery.

a The cell shape in primary PSCs and less than 15 passages PSCs (Original magnification, 10×). **b** The expressions of RB1CC1, ULK1, LC3, P62 and α-SMA were explored in primary PSCs, activated primary PSCs, PSCs and activated PSCs via Western Blot assays. GAPDH served as the internal control. The results are representative of three independent experiments.

Supplementary Fig. 2 The TGF-β dose- and time- dependent changes of PSCs activation, RB1CC1 expression and autophagy activation.

a The expressions of RB1CC1, ULK1, p-ULK1, P62, α-SMA and LC3 Ⅱ/Ⅰ in four TGF-β concentrations (0, 5ng/ml, 10ng/ml and 20ng/ml). **b** The expressions of RB1CC1, ULK1, p-ULK1, P62, α-SMA and LC3 Ⅱ/Ⅰ in four time points (0, 12h, 24h and 48h) with the concentrations of 10ng/ml. GAPDH served as the internal control. The results are representative of three independent experiments.

Supplementary Fig. 3 The expressions of ULK1, Collagen Ⅰ and P62 in CQ-treated and Rapamycin-treated groups.

a The relative expressions of ULK1, Collagen Ⅰ and P62 in negative control, TGF-β, CQ and CQ plus TGF-β groups. **b** The relative expressions of ULK1, Collagen Ⅰ and P62 in negative control, TGF-β, Rapamycin and Rapamycin plus TGF-β groups. The

relative expression represents the ratio of target to GAPDH. Data are expressed as mean ± SD. The results are representative of three independent experiments. **(*$p<0.05$, **$p<0.01$ and ns $p>0.05$)**

Supplementary Fig. 4 Establishment of knockdown and conduct of designing siRNA and shRNA targeting RB1CC1.

a The expressions of RB1CC1 in control and si-RB1CC1 groups. **b and c** The RB1CC1 protein and mRNA expressions were tested in different groups. The sh-RB1CC1-1 was the optimum selection. Data are expressed as mean ± SD. The results are representative of three independent experiments. **(**$p<0.01$, *** $p<0.001$ and ns $p>0.05$)**

Supplementary Fig. 5 RB1CC1 inhibits PSCs cell viability and proliferation.

a and b Knockdown of RB1CC1 promoted PSCs cell viability (Bars =500μm). **c and d** The proliferation and DNA duplications of PSCs were determined via CCK-8 and EdU assays in negative control, TGF-β-treated, RB1CC1-overexpressed and RB1CC1 overexpressed plus TGF-β-treated groups (Bars =500μm). Data are expressed as mean ± SD. The results are representative of three independent experiments. **(*** $p<0.001$ and ns $p>0.05$)**

Supplementary Fig. 6 Knockdown of RB1CC1 suppresses PSCs apoptosis and downregulation of RB1CC1 could also reverse TGF-β-induced cell apoptosis. The results are representative of three independent experiments.

Supplementary Fig. 7 Downregulation of RB1CC1 suppresses PSCs migration and it could abolish TGF-β-induced cell migration. Data are expressed as mean ± SD. The results are representative of three independent experiments. (* $p<0.05$ and ** $p<0.01$)

Supplementary Fig. 8 TGF-β promotes the synthesis of matrix met alloproteinases (MMPs). Inhibition of RB1CC1 downregulates the expressions of MMP-2 and MMP-9 and it could also weaken the TGF-β-induced MMPs expression. The results are representative of three independent experiments.

Supplementary Fig. 9 The semi-quantitation of RB1CC1, ULK1, p-ULK1, α-SMA，P62 and LC3 in eight different treated groups.

a The relative expressions of RB1CC1, ULK1, p-ULK1, α-SMA, P62 and LC3 in negative control, TGF-β, CQ and CQ plus TGF-β groups. **b** The relative expressions of RB1CC1, ULK1, p-ULK1, α-SMA, P62 and LC3 in negative control, TGF-β, Rapamycin and Rapamycin plus TGF-β groups. The relative expression represents the ratio of target to GAPDH. Data are expressed as mean ± SD. The results are representative of three independent experiments. **(*$p<0.05$, **$p<0.01$, ***$p<0.001$ and ns $p>0.05$)**

Supplementary Fig. 10 The predicted three binding sites of RB1CC1 in ULK1

promotor.

Supplementary Fig. 11 The effects of sliencing or overexpressing RB1CC1 in pancreatic acinar cells and endocrine cells.

a The expressions of RB1CC1, ATG5, ATG7, ULK1, p-ULK1, P62 and LC3 in negative control, RB1CC1 knockdown, control and RB1CC1 overexpression groups in NIT-1 cell lines. **b** The expressions of RB1CC1, ATG5, ATG7, ULK1, p-ULK1, P62, LC3 and TGF-β in negative control, RB1CC1 knockdown, control and RB1CC1 overexpression groups in MPC-83 cell lines. The results are representative of three independent experiments.
