# Supplementary material for: RB1CC1-enhanced autophagy facilitates PSCs activation and pancreatic fibrogenesis in chronic pancreatitis
Source: Cell Death Dis. 2018 Sep 20;9(10):952. doi: 10.1038/s41419-018-0980-4 (PMC6147947; doi:10.1038/s41419-018-0980-4)

Supplementary Figure 1

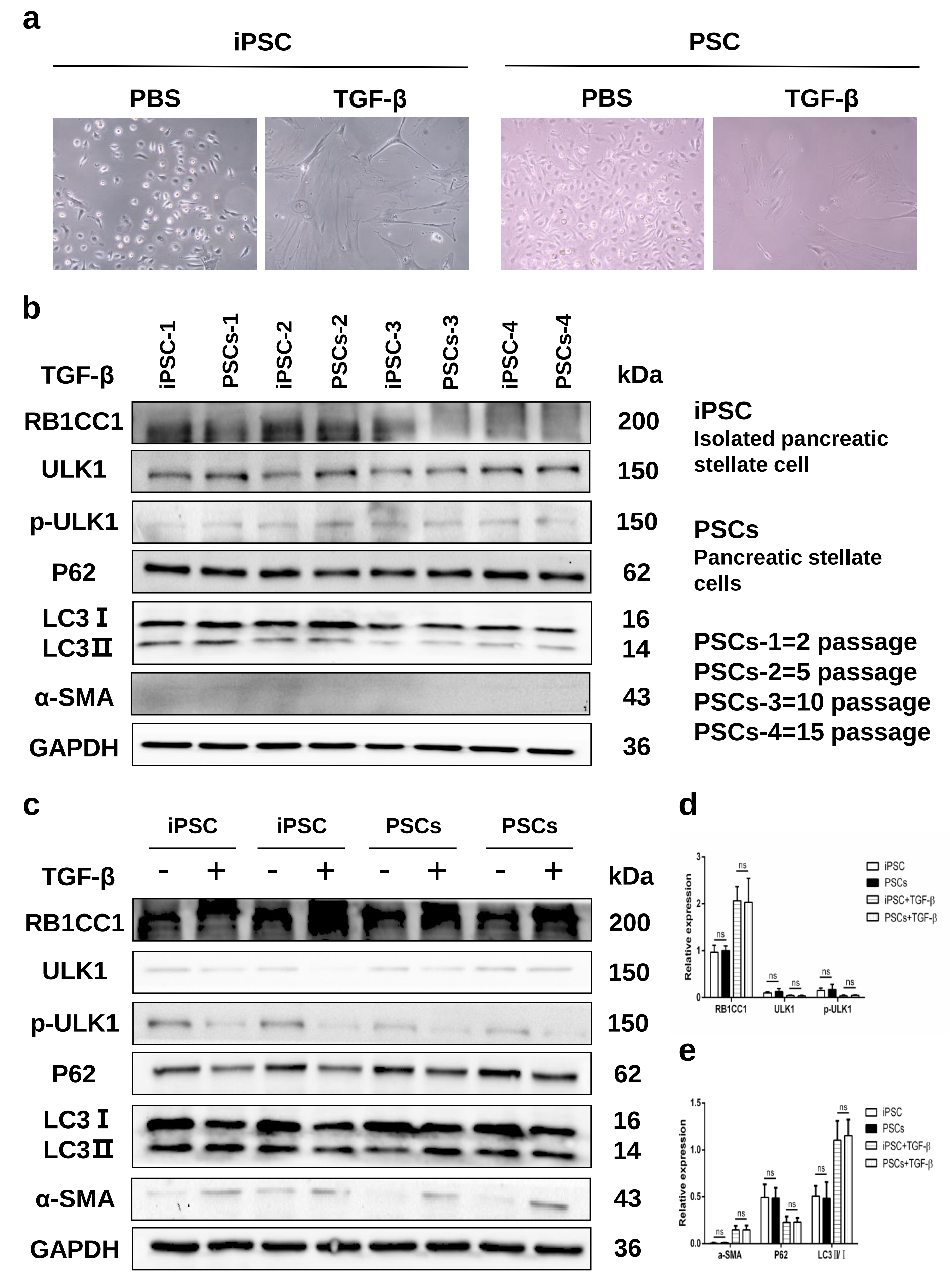

Supplementary Figure 2

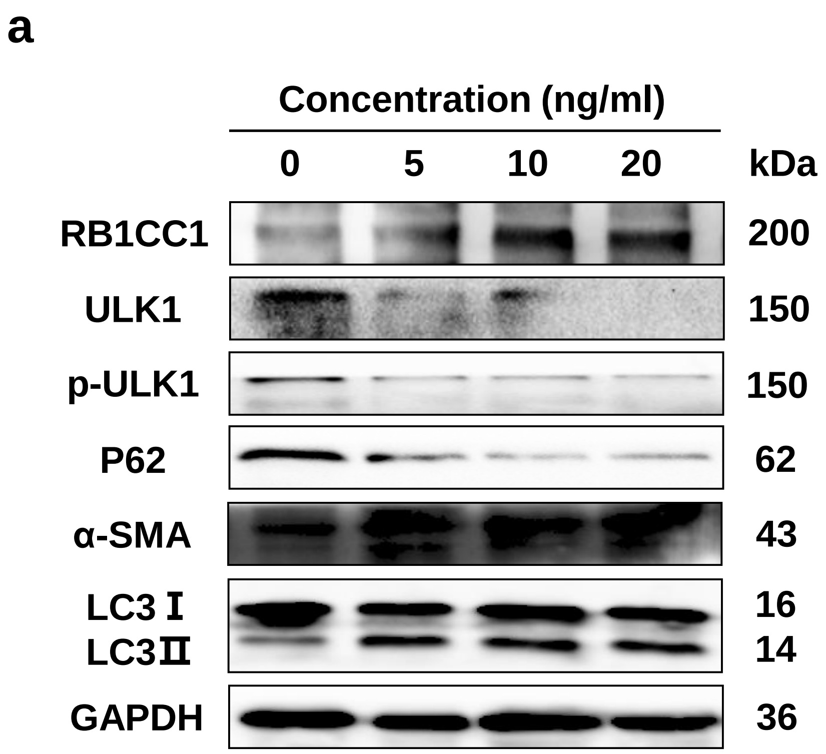

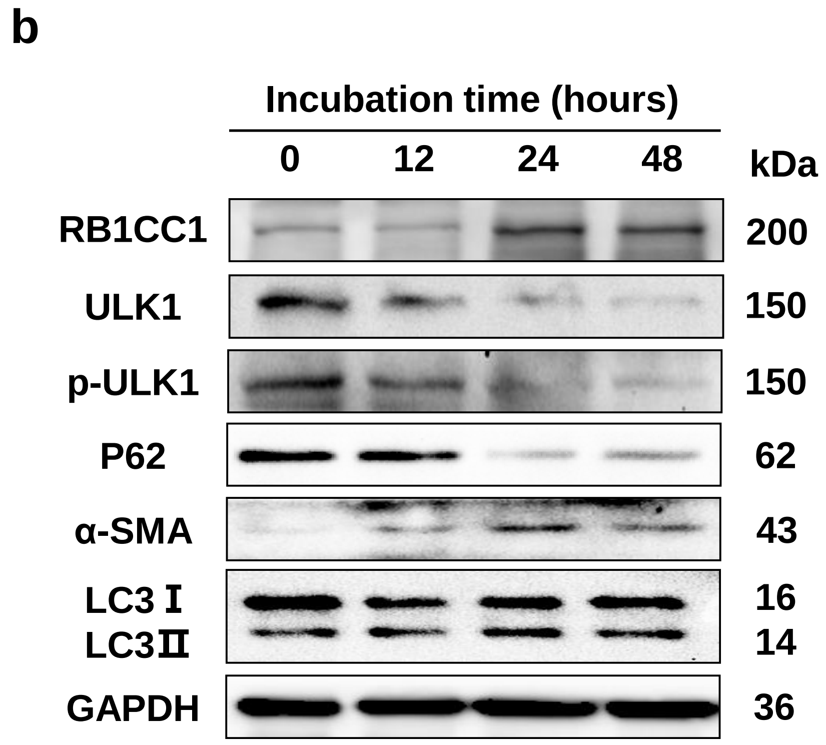

Supplementary Figure 3

a

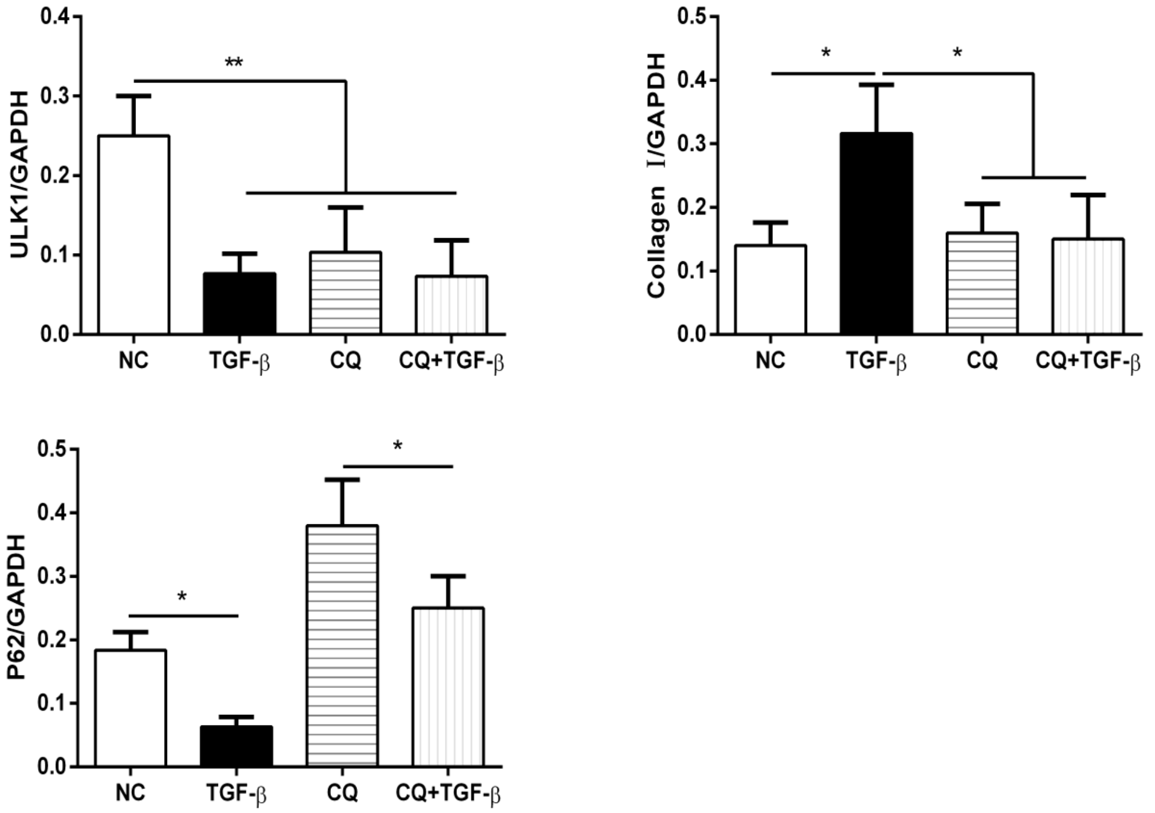

b

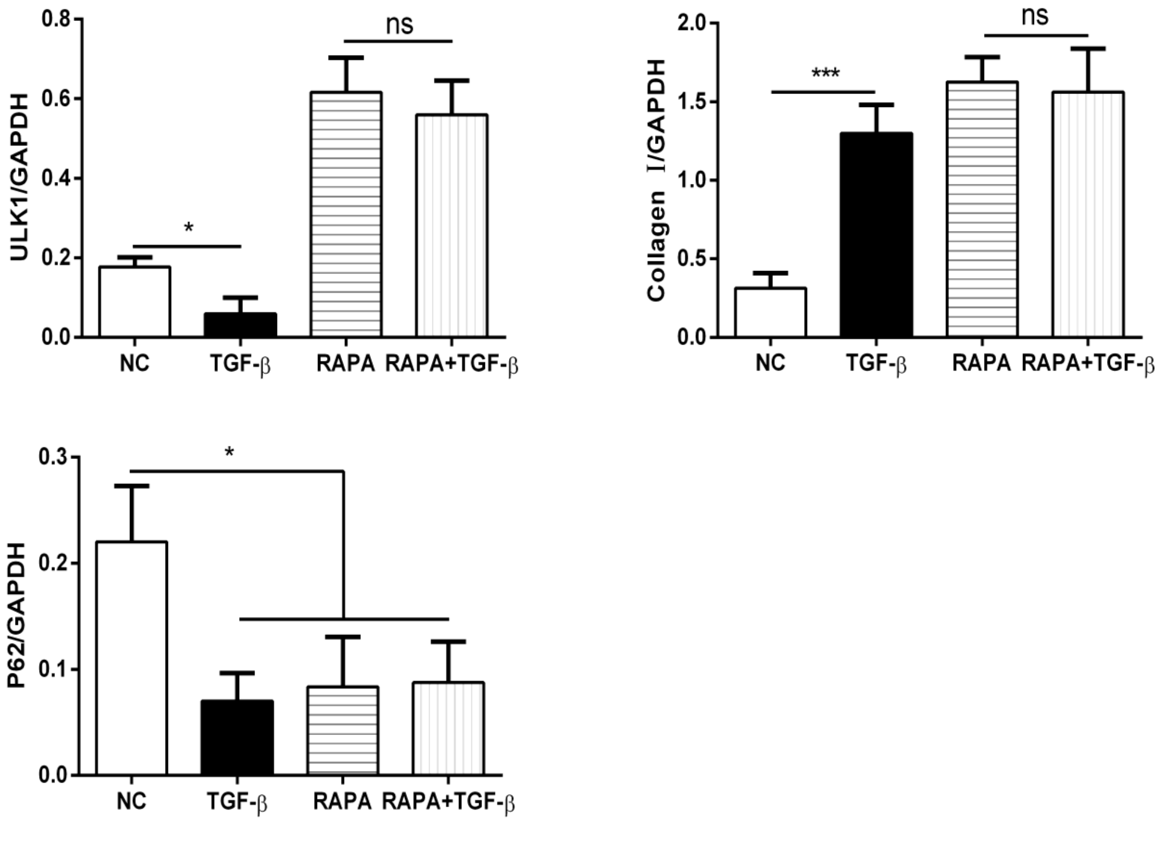

Supplementary Figure 4

a

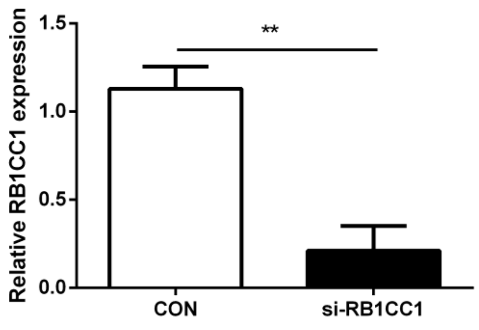

b

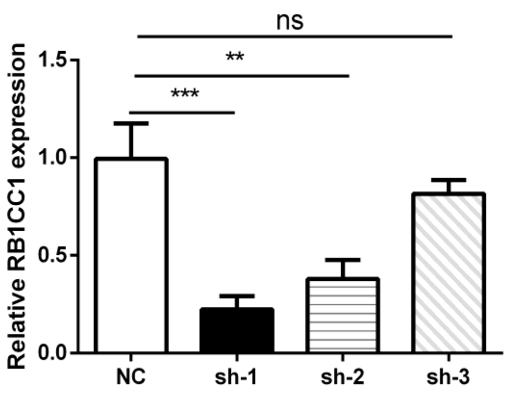

c

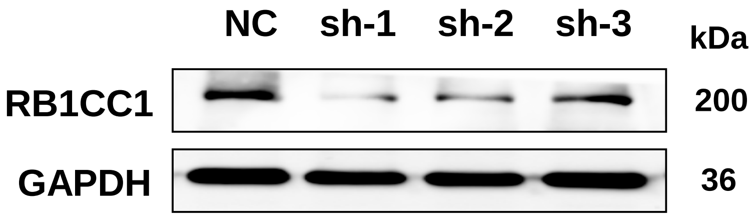

Supplementary Figure 5

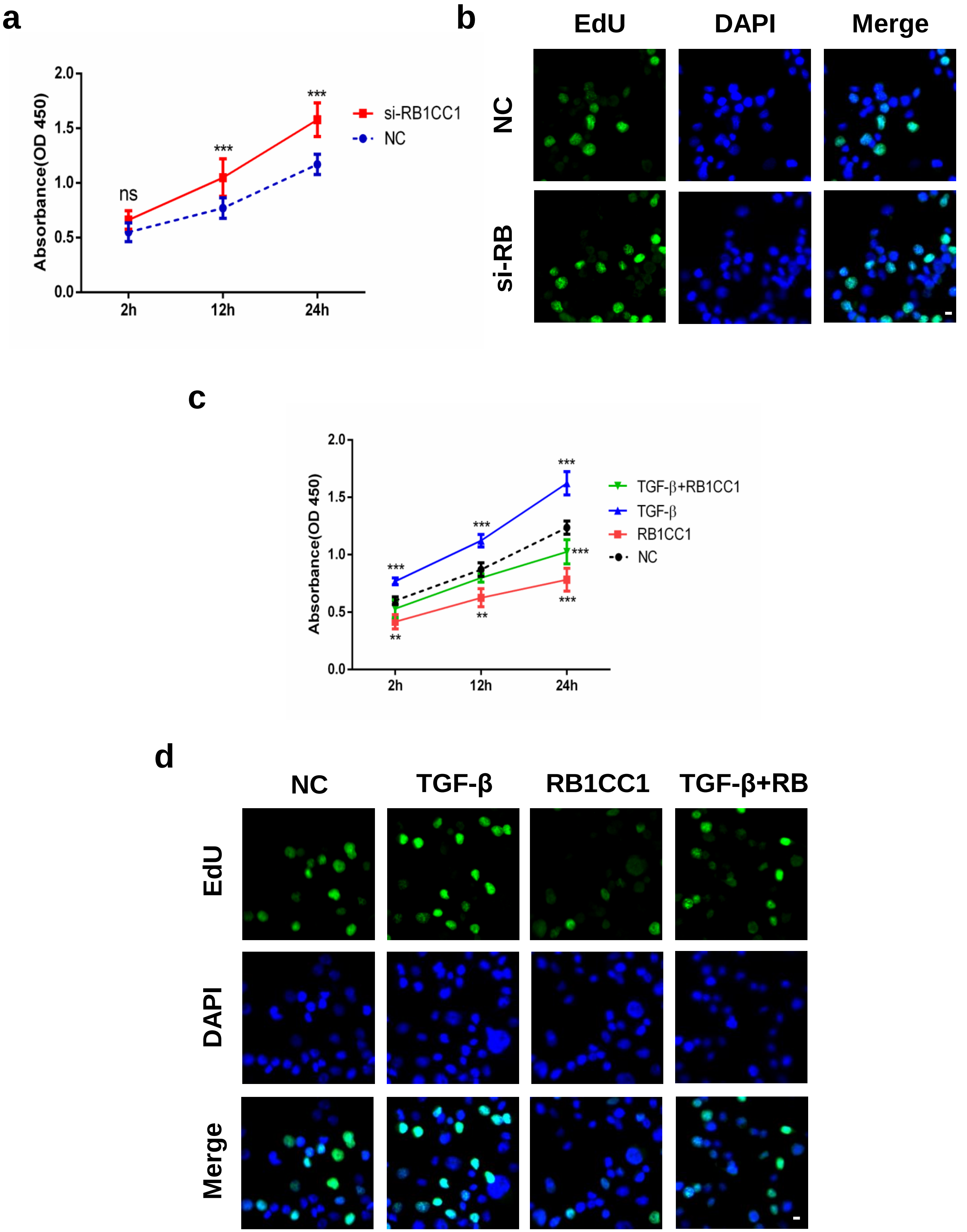

Supplementary Figure 6

a

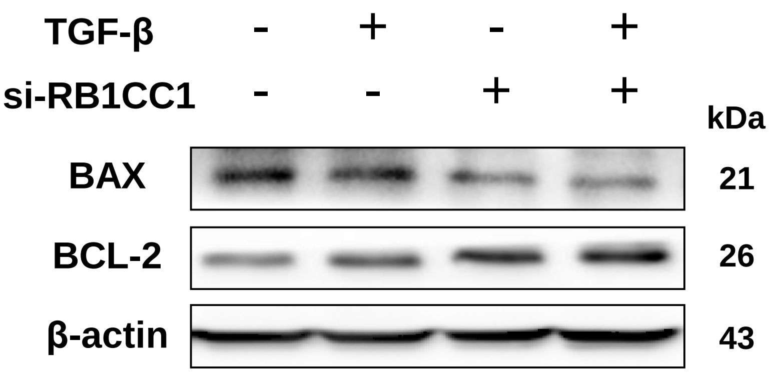

Supplementary Figure 7

a

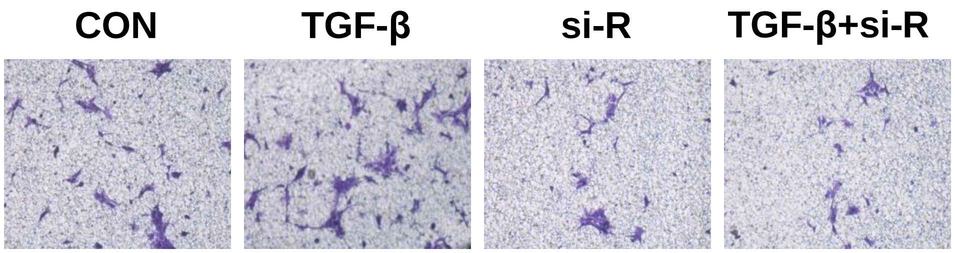

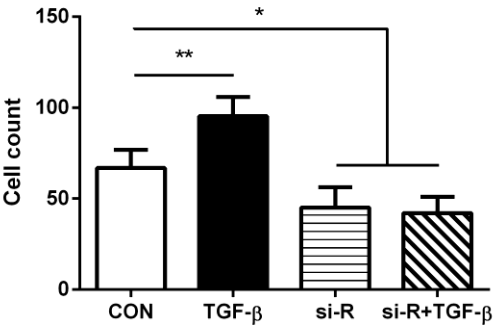

Supplementary Figure 8

a

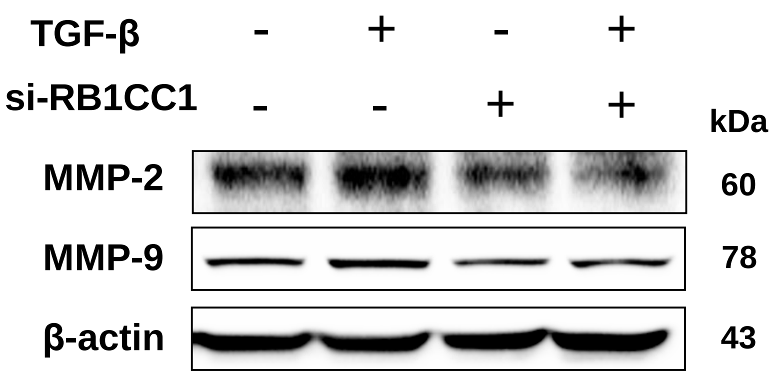

Supplementary Figure 9

a

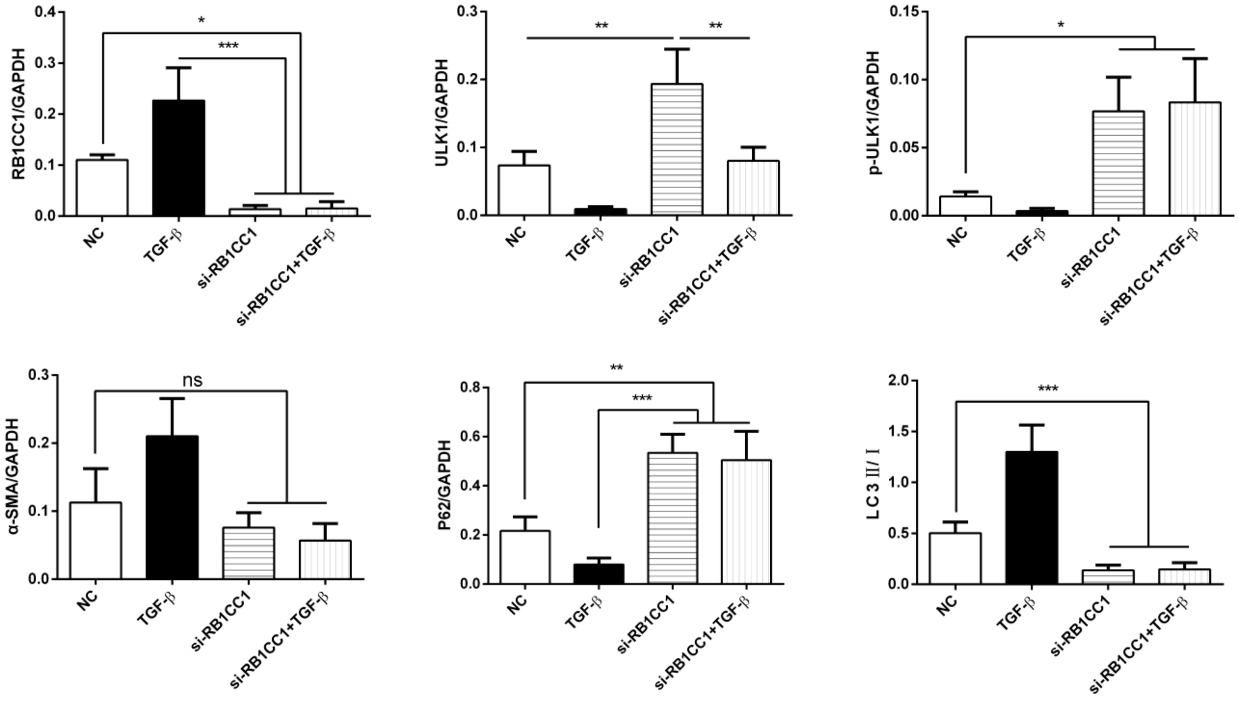

b

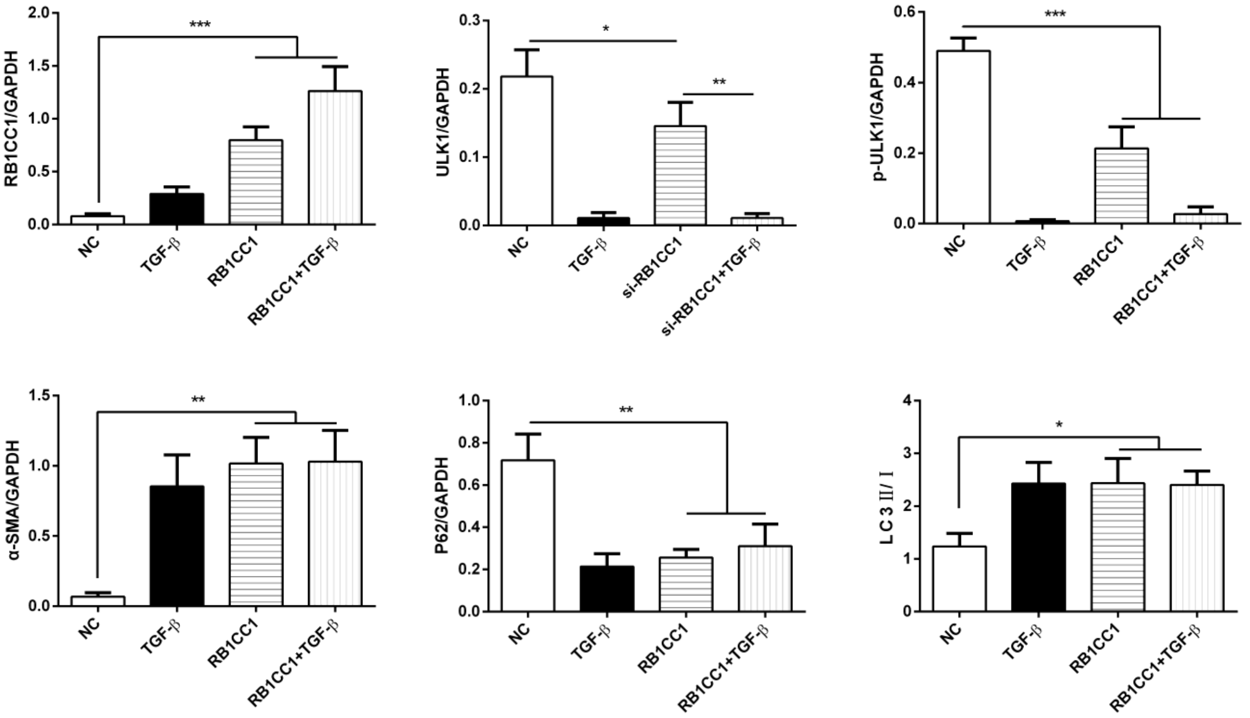

Supplementary Figure 10

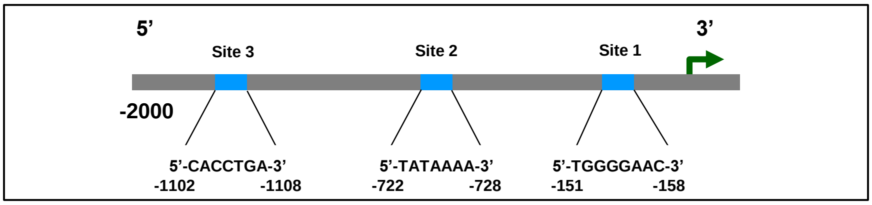

Supplementary Figure 11

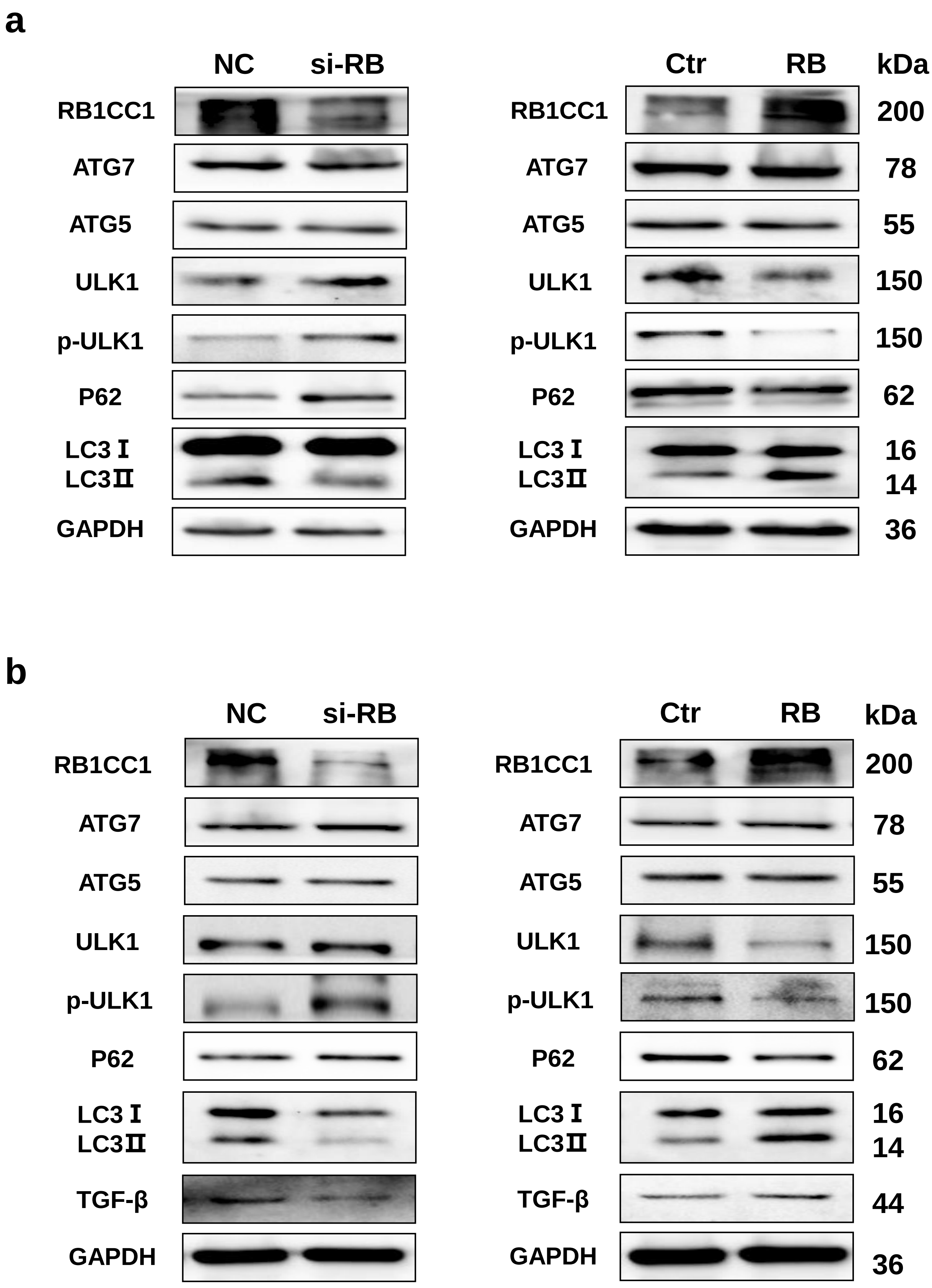

Supplement: Supplementary file 2 — Revised Supplementary Figure [file 41419_2018_980_MOESM2_ESM.pdf]
