# Supplementary material for: RB1CC1-enhanced autophagy facilitates PSCs activation and pancreatic fibrogenesis in chronic pancreatitis
Source: Cell Death Dis. 2018 Sep 20;9(10):952. doi: 10.1038/s41419-018-0980-4 (PMC6147947; doi:10.1038/s41419-018-0980-4)
Supplement: Supplementary file 5 — Supplementary Table 3 [file 41419_2018_980_MOESM5_ESM.docx]

Table 3. Characteristics of pancreas donors of normal controls and CP patients.

| **Donors** | **Age（years）** | **Sex** | **Pathological Diagnosis** | **Surgical Procedure** |
| --- | --- | --- | --- | --- |
| **Normal control 1** | 49 | Male | Pancreatic cancer | Pancreaticoduodenectomy |
| **Normal control 2** | 50 | Male | Cholangiocarcinoma | Pancreaticoduodenectomy |
| **Normal control 3** | 44 | Female | Solid pseudopapillary neoplasm of the pancreas | Distal pancreatectomy |
| **Normal control 4** | 62 | Male | Pancreatic cancer | Pancreaticoduodenectomy |
| **Normal control 5** | 64 | Male | Cholangiocarcinoma | Pancreaticoduodenectomy |
| **Normal control 6** | 50 | Male | Carcinoma of duodenal papilla | Pancreaticoduodenectomy |
| **Normal control 7** | 36 | Female | Solid pseudopapillary neoplasm of the pancreas | Distal pancreatectomy |
| **Normal control 8** | 45 | Male | Pancreatic cancer | Distal pancreatectomy |
| **Normal control 9** | 42 | Male | Carcinoma of duodenal papilla | Pancreaticoduodenectomy |
| **Normal control 10** | 58 | Male | Cholangiocarcinoma | Pancreaticoduodenectomy |
| **Normal control 11** | 40 | Female | Solid pseudopapillary neoplasm of the pancreas | Distal pancreatectomy |
| **Normal control 12** | 45 | Female | Cystadenoma of pancreas | Distal pancreatectomy |
| **Normal control 13** | 56 | Male | Pancreatic cancer | Pancreaticoduodenectomy |
| **Normal control 14** | 58 | Male | Pancreatic cancer | Distal pancreatectomy |
| **Normal control 15** | 45 | Male | Pancreatic cancer | Distal pancreatectomy |
| **CP patient 1** | 47 | Male | Chronic pancreatitis | Frey procedure |
| **CP patient 2** | 49 | Male | Chronic pancreatitis | Duodenum-preserving pancreatic head resection |
| **CP patient 3** | 56 | Male | Chronic pancreatitis | Pancreaticoduodenectomy |
| **CP patient 4** | 62 | Male | Chronic pancreatitis | Choledochojejunostomy |
| **CP patient 5** | 42 | Male | Chronic pancreatitis | Partington procedure |
| **CP patient 6** | 55 | Male | Chronic pancreatitis | Choledochojejunostomy |
| **CP patient 7** | 50 | Male | Chronic pancreatitis | Pancreaticoduodenectomy |
| **CP patient 8** | 49 | Male | Chronic pancreatitis | Partington procedure |
| **CP patient 9** | 62 | Male | Chronic pancreatitis | Partington procedure |
| **CP patient 10** | 55 | Female | Chronic pancreatitis | Pancreaticoduodenectomy |
| **CP patient 11** | 42 | Male | Chronic pancreatitis | Frey procedure |
| **CP patient 12** | 44 | Male | Chronic pancreatitis | Partington procedure |
| **CP patient 13** | 49 | Male | Chronic pancreatitis | Choledochojejunostomy |
