# Supplementary material for: RB1CC1-enhanced autophagy facilitates PSCs activation and pancreatic fibrogenesis in chronic pancreatitis
Source: Cell Death Dis. 2018 Sep 20;9(10):952. doi: 10.1038/s41419-018-0980-4 (PMC6147947; doi:10.1038/s41419-018-0980-4)
Supplement: Supplementary file 6 — Supplementary Table 4 [file 41419_2018_980_MOESM6_ESM.docx]

Table 4. Characteristics of plasma donors of healthy volunteers and CP patients.

| **Donors** | **Age（years）** | **Sex** | **Pathological Diagnosis** |
| --- | --- | --- | --- |
| **Healthy volunteer 1** | 56 | Male | NA |
| **Healthy volunteer 2** | 49 | Male | NA |
| **Healthy volunteer 3** | 52 | Male | NA |
| **Healthy volunteer 4** | 44 | Female | NA |
| **Healthy volunteer 5** | 57 | Male | NA |
| **Healthy volunteer 6** | 46 | Male | NA |
| **Healthy volunteer 7** | 40 | Female | NA |
| **CP patient 1** | 47 | Male | Chronic pancreatitis |
| **CP patient 2** | 49 | Male | Chronic pancreatitis |
| **CP patient 3** | 56 | Male | Chronic pancreatitis |
| **CP patient 4** | 62 | Male | Chronic pancreatitis |
| **CP patient 5** | 42 | Male | Chronic pancreatitis |
| **CP patient 6** | 55 | Male | Chronic pancreatitis |
| **CP patient 7** | 50 | Male | Chronic pancreatitis |
| **CP patient 8** | 49 | Male | Chronic pancreatitis |
| **CP patient 9** | 62 | Male | Chronic pancreatitis |
| **CP patient 10** | 55 | Female | Chronic pancreatitis |
| **CP patient 11** | 42 | Male | Chronic pancreatitis |
